# Sales characteristics of Pokémon trading cards: A prospective one-year field study

**DOI:** 10.1371/journal.pone.0334289

**Published:** 2026-03-12

**Authors:** Johannes Heck, Sarah Schormann, Benjamin Krichevsky, Adrian Groh, Thorben Pape, Sebastian Schröder, Alexander Glahn, Carsten Schumacher, Martin Schulze Westhoff

**Affiliations:** 1 Department of Psychiatry, Social Psychiatry and Psychotherapy, Hannover Medical School, Hannover, Germany; 2 Institute for General Practice and Palliative Care, Hannover Medical School, Hannover, Germany; 3 Department of Information and Communication, Faculty III, University of Applied Sciences and Arts, Hannover, Germany; 4 secuTrial, Berlin, Germany; 5 Interdisciplinary Emergency Department, University Hospital Schleswig-Holstein – Campus Kiel, Kiel, Germany; 6 Department of Respiratory Medicine and Infectious Diseases, Hannover Medical School, Hannover, Germany; 7 PRACTIS Clinician Scientist Program, Dean’s Office for Academic Career Development, Hannover Medical School, Hannover, Germany; 8 Center for Clinical Trials, Hannover Medical School, Hannover, Germany; The University of Electro-Communications, JAPAN

## Abstract

Since its inception in North America in 1999, the Pokémon trading card game has become a global phenomenon, combining strategic gameplay with collectible culture. The economic value of Pokémon trading cards, particularly as collector’s items, underscores their significance beyond mere playthings, making them a subject of both academic and commercial interest. In the present study, a convenience sample of 300 Pokémon trading cards were offered on the e-commerce platform eBay between 28 May 2024 and 27 May 2025, with a minimum follow-up period of 3 months per card. All cards were sold nationally in Germany to avoid high shipping costs for international transactions. The aim of the study was to comprehensively analyze sales characteristics of Pokémon trading cards, using both descriptive and inferential statistical techniques including “survival” analyses and Cox regression models. 73.3% (220/300) of the trading cards were sold during the study period, generating a total revenue of 923.60 €. Sales prices showed a markedly skewed distribution, with a median of 1.95 € per trading card. Rare and holofoil cards contributed overproportionately to the total revenue. Uncommon cards exhibited an interesting economic potential due to rapid sales kinetics. Cards from the expansions Team Rocket and Gym Challenge were considerably more popular than Base Set 2 cards, as reflected by shorter “survival” times. The vast majority of buyers were male, but female buyers spent more money per trading card on average. The German federal states Thuringia and Hamburg emerged as Pokémon trading card hubs, with numbers of cards sold and cumulated revenues exceeding expectations based on population sizes.

## Introduction

Inspired by its creator Satoshi Tajiri’s passion for collecting insects, the Pokémon game has developed into one of the largest and most profitable multimedia franchises of the world since its inception in 1996, comprising an animated television (TV) series, movies, videogames, as well as a trading card game (TCG) [1–8]. The word Pokémon is an abbreviated version of the term “pocket monsters” (Japanese original *Poketto Monsuta*), which describes the creatures that populate the Pokémon fantasy world, and which have to be caught, trained, and sent to non-lethal combat against other Pokémon by players (so-called Pokémon Trainers) [1,2,8].

The Pokémon TCG combines competitive, strategic gameplay with collectible culture [9], with trading cards appealing to both players and collectors, and also across generations [10]. Apart from the TCG primary market, on which booster packs (i.e., sealed physical packs containing a defined number of random cards of varying rarity [11]) are sold, there also exists a lively secondary market for TCGs, where players can directly purchase the one card they are looking for [9].

The Pokémon TCG is a two-player, turn-based card game in which each participant assumes the role of a Pokémon Trainer [6,7]. Players assemble decks of 60 cards, comprising three primary types: Pokémon, Energy, and Trainer cards [6,7]. Pokémon cards represent the creatures themselves, each with unique abilities and forms of attack [6,7]. Energy cards provide the resources required for Pokémon to execute attacks, while Trainer cards offer various strategic effects, such as drawing additional cards or disrupting the opponent’s strategy [6,7]. Gameplay revolves around deploying Pokémon from a “bench” to the active position to engage in combat with the opponent’s Pokémon, and strategically evolving Pokémon into more powerful forms [6,7]. The objective is to knock out all of the opponent’s Pokémon (i.e., reduce their health points to zero) [6,7]. The player who achieves this goal wins the match [7].

Between 1999 and 2003, the Pokémon TCG was designed by Wizards of the Coast, the publisher of Magic the Gathering, another highly popular TCG [7–9]. In June 2003, Nintendo took over the Pokémon TCG license, and transferred design and development to Pokémon USA, one of its subsidiaries [7,9]. Over the years, numerous expansions (“sets”) have been added to the Pokémon TCG, each introducing new Pokémon, gameplay mechanics, and artwork [7].

From around the mid-2000s onwards, the public interest in Pokémon declined [3,12], but amid the coronavirus disease 2019 (COVID-19) pandemic, influencers and streamers gave the Pokémon TCG an unexpected revival [4,13]. Some card deals executed during that period fetched record-breaking sums [4,13–17]. For example, a first-edition holographic shadowless Charizard card sold for US$369,000 in 2020 [14,18]. The arguably most famous and most valuable collector’s item in the Pokémon world is the Pikachu Illustrator card [14,19,20]. It was awarded in Japan in 1997 to the winners of an art contest [19]. Of the 39 copies originally produced, only a few still exist today, explaining their enormous value [19–21]. In February 2021, one copy sold for US$375,000 on eBay [14,19]. One year later, in April 2022, a nearly flawless Pikachu Illustrator copy broke all records when YouTuber and wrestling star Logan Paul purchased the card for US$5.275 million [14,15,19,21]. Recently, in an auction conducted by the auction firm Goldin on 16 February 2026, Paul was able to resell said card for the astronomical price of US$16.492 million (together with the diamond necklace he wore to present the card to the public for the first time during his World Wrestling Entertainment (WWE) debut at WrestleMania 38 in 2022) [15].

Reportedly, as of March 2023 there had been almost 53 billion cards produced across 14 different languages and 89 countries [4]. Two years later, in March 2025, this number had risen to over 75 billion cards [7], “enough to wrap around Earth end-to-end 165 times” as Preston Fore recently calculated in an article in Fortune [17].

Besides the spectacular Pokémon trading card exemplars cited above [14,15,19,21], there exists also a large secondary market for trading cards of comparatively lower value. To play the Pokémon TCG, as well as to complete a specific set, also lower-value cards are required by players and collectors alike, exemplified by the tension-creating slogan of the Pokémon franchise “Gotta catch ‘em all!” [22,23]. The economic value of Pokémon trading cards is therefore a subject of considerable interest, both from a commercial and scientific viewpoint.

Non-professional sellers on the TCG secondary market are confronted on a regular basis with the difficulty of accurately and realistically valuating their items. International companies such as PSA (Professional Sports Authenticator) [24] and BGS (Beckett Grading Services) [25] offer special evaluation services known as grading for collectibles like Pokémon trading cards [5,14,26–28]. Graded cards are authenticated and encapsulated in plastic cases to preserve their condition [5,26]. The quality of the cards is evaluated on a numerical scale from 1 to 10, with 10 describing a card in perfect condition [5,14,26–28]. Professional grading of cards increases their resell value but costs about US$25 per item; hence it is only economically reasonable for high-value cards [26].

The primary goal of our study was to comprehensively analyze sales characteristics of Pokémon trading cards, taking card characteristics as well as buyers’ demographics into consideration. In addition, the present study sought to explore how the recent advent of artificial intelligence (AI) tools might potentially aid non-professional sellers on the TCG secondary market in estimating sales prices for their items. To this end, the sales prices of a convenience sample of 300 Pokémon trading cards across different sets were estimated from the user’s perspective with the aid of the AI tool Perplexity [29] and subsequently offered on the e-commerce platform eBay, adopting a posted-price strategy. eBay has been widely used previously in economic research to conduct field experiments [10,28,30–39].

## Materials and methods

### Ethical considerations

The focus of the present study was economic; medical data were only obtained from the literature database PubMed/MEDLINE as part of a literature review for the Discussion. Our primary study subject were sales transactions on the e-commerce platform eBay. Sales transactions data relevant to the purpose of this study (i.e., card details and buyer demographics) were matched automatically by eBay as part of the purchase process and were extracted in anonymized form into a Microsoft Excel spreadsheet immediately after a sales transaction had been completed (details are provided in the chapters “Study design” and “Buyer demographics”). No clinical trial, non-interventional study, survey or questionnaire involving human participants were conducted. Still, since eight out of nine authors of this article are medical doctors, the Declaration of Helsinki and its amendments (latest version dating from October 2024) [40] were taken into consideration, but were found to be not applicable to the present setting because no medical research (besides a review of the literature) was performed. As required by the *Berufsordnung der Ärztekammer Niedersachsen* (Professional Code of Conduct of the Medical Association of Lower Saxony) [41], the local Ethics Committee (i.e., the Ethics Committee of Hannover Medical School) was contacted and asked whether an evaluation of anonymized data required a formal consultation and ethics vote. According to the Ethics Committee of Hannover Medical School, this was not the case, and a formal ethics vote was waived. Since the present study did not involve human participants, an informed consent process was not applicable.

### Study design

The study was devised as a prospective field study encompassing a study period of one year. Between 28 May 2024 and 27 May 2025, a convenience sample of 300 privately owned Pokémon trading cards were offered on the e-commerce platform eBay [42] via a personal account with a minimum follow-up period of 3 months per card (S1 Dataset). Since to the best of our knowledge, no previous studies had dealt with the topic of sales characteristics of Pokémon trading cards in a comparable fashion, no formal sample size calculation was conducted. All cards were sold nationally in Germany to avoid high shipping costs for international transactions [26]. National shipping costs lay between 0.85 € (standard letter up to 20 grams) and 1.95 € (standard letter with priority shipping) [43], and were paid by the buyers. Shipping costs were excluded from the analysis.

The primary goal of our study was to comprehensively analyze sales characteristics of Pokémon trading cards, taking card characteristics as well as buyers’ demographics into consideration. An exploratory research goal was to assess the potential utility of the AI tool Perplexity [29] as a decision support tool from the user’s perspective for estimating realistic sales prices of Pokémon trading cards, when adopting a posted-price strategy. For all 300 cards, Perplexity was provided with the relevant card information (i.e., card name, set, presence/absence of holographic effects, card condition, etc.) and was prompted to suggest a market price range. The same prompt (in German language) was used for all cards: *“Welche Preisspanne kann aktuell auf eBay beim Verkauf einer Pokémon-Sammelkarte [X] in [Y] Zustand erzielt werden?“* (“What price range can currently be achieved on eBay when selling a Pokémon trading card [X] in [Y] condition?”), with X and Y specifying the abovementioned card information and the card condition (e.g., “very good”), respectively. All market price suggestions by Perplexity were manually cross-checked on eBay for feasibility before a final decision about the posted price was made (within, below or above the price range indicated by Perplexity). Hence, while Perplexity was used as a decision support tool, the final decision about posted prices resided with the human seller. While eBay auctions were not conducted in the present study, price offers by potential buyers were allowed for all cards [44].

As the Pokémon trading cards investigated in this study had been obtained by JH and MSW during their childhood/adolescence in the late 1990s and early 2000s, respectively, previous purchase prices are not available. Hence, in the present study, revenues instead of profits are analyzed.

### Pokémon trading card layout

The Pokémon trading cards investigated in this study featured a similar layout (S2 Table). The most important elements of the cards (for the purpose of this study) are [45]:

- Card name (displayed at the very top of the card)
- Illustration (located centrally on the card): may contain a holographic effect (see section “Holographic effects” for details)
- Set symbol (located at the bottom right next to the illustration): indicates which expansion a card belongs to, e.g., a flower representing the Jungle set. Of note, Base Set cards do not feature a set symbol.
- Set information (located in the bottom right corner of the card): shows the card number out of the set (e.g., 4/102 refers to the fourth card of the Base Set, which contains 102 cards in total).
- Rarity symbol (see section “Rarity” for details)

### Card type

Trading cards were categorized as [45]:

- Pokémon cards
- Trainer cards
- Energy cards

### Sets

In our sample, Pokémon trading cards from nine different sets were contained:

- Base Set: First released in Japan in October 1996, the Base Set is the foundational set of the Pokémon TCG, featuring a total of 102 cards. The English version followed in January 1999, while the launch of the German version was in December 1999 [46,47].
- Jungle: The Jungle set expanded the original Pokémon TCG with 64 cards, first appearing in Japan in March 1997. The English release came in June 1999, and the German version followed in February 2000 [12,48].
- Fossil: The Fossil set was released in Japan in June 1997 (the launch of the English and German versions was in October 1999 and in April 2000, respectively), adding another 62 cards to the Pokémon TCG [12,49].
- Team Rocket: Launched in Japan in November 1997, this set introduced Dark Pokémon—Pokémon that reappeared in their evil form [12]—across 83 cards (82 regular cards plus 1 secret card (i.e., a card whose number is greater than the listed number of cards in the set [50])) into the TCG [7,51]. The English and German versions followed in April and July 2000, respectively [51].
- Gym Heroes: Introducing Owner’s Pokémon (i.e., Pokémon that have a Trainer’s name [52]), Gym Heroes is a 132-card collection which first released in Japan in October 1998 [7,53]. The English version launched in August 2000 [53]. A German version does not exist [53,54].
- Gym Challenge: This set debuted in Japan in June 1999, adding another 132 cards to the Pokémon TCG [55]. The English version followed in October 2000 but did not receive an official German translation [55,56].
- Black Star Promo: Between July 1999 and March 2003, 53 promotional cards for special events such as tournaments or movie premieres were released by Wizards of the Coast [57,58], commonly referred to as (Wizards) Black Star Promo cards [57,59]. Twenty-four of those 53 cards were translated to German [59].
- Base Set 2: The Base Set 2 is a 130-card collection composed of reprints of the Base Set and Jungle set [60,61]. It was released exclusively in English in February 2000; a Japanese equivalent does not exist, nor was the set translated to German [60,61].
- Neo Genesis: Whereas the aforementioned sets exclusively featured Generation I Pokémon [46,48,49,51,53,55], the Neo Genesis set was the first to introduce Generation II Pokémon into the TCG [62]. The Neo Genesis set comprises 111 cards and debuted in Japan in February 2000. The English release followed in December of the same year, while the German version launched in February 2001 [62].

### Rarity

In this study, the basic three-level rarity classification of Pokémon trading cards was used [10,63–66] (see also S2 Table):

- Common (circle symbol)
  - Uncommon (diamond symbol)
- Rare (star symbol)

In the following, rarity categories shall be written in capitalized form to differentiate them from the corresponding colloquial expressions.

### Holographic effects

Trading cards were categorized as holofoil or non-holofoil (matte) cards [10,65]. Holofoil cards are characterized by a shiny, reflective, rainbow-like effect residing over the main Pokémon illustration (refer, for example, to the Glurak (English name: Charizard) 4/102 card in S2 Table).

### Language

The Pokémon trading cards contained in the analyzed sample were in German, English, Japanese or Italian language. For statistical reasons—and because all cards were sold nationally to buyers residing in Germany—English, Japanese and Italian were grouped together as non-German languages.

### Editions

Editions were differentiated into first edition or unlimited edition [10,66]. First-edition cards refer to those that are printed in the first print run of a particular set, only being available in booster packs for a limited period of time after the initial release of a particular set [67]. They are later replaced by an unlimited edition until the printing of that set is discontinued [67].

### Card condition

To describe the condition of Pokémon trading cards, eBay’s 4-tier classification scheme for trading cards was used (listed from best to worst condition) [68]:

- Near Mint or Better: indicating minimal or no visible wear
- Excellent: slight signs of wear, minimal flaws
- Very Good: moderate wear, some noticeable flaws
- Poor: extensive wear, noticeable flaws and damage

Card condition was assessed by a single evaluator (JH), taking the following aspects into consideration:

- Surface (front and back): presence/absence of scratches, creases, stains or scuffs
- Edges: evaluation of potential whitening or fraying
- Corners: should ideally be sharp and undamaged; bent or chipped corners lower a card’s condition
- Centering: the degree of alignment of the printed image within the card’s borders

Of note, the cards were not evaluated independently by a professional grading service such as PSA [24] or BGS [25]. Similar to rarity categories, condition grades shall be written in capitalized form to differentiate them from the corresponding colloquial expressions.

### Print accuracy

Pokémon trading cards were classified as correctly printed cards or as error cards (misprints) [69].

### Buyer demographics

All data on buyers, which were provided by eBay during sales transactions (i.e., first name, family name, shipping address), were analyzed in anonymized form. Since no information on buyers’ gender self-identification was available, buyers’ first names were entered into an online compendium containing over 87,000 first names from German-speaking regions [70]. If the compendium indicated that a buyer’s first name was typically male (e.g., “Thomas”) or typically female (e.g., “Sandra”), the buyer was considered male or female, respectively [70]. If only the initial of the first name was provided by eBay (e.g., “L.”), gender was considered as “not determinable”. Values belonging to the gender category “not determinable” were excluded from inferential statistics that investigated potential gender-specific differences.

The Federal Republic of Germany consists of 16 federal states, three of which are so-called city-states (Berlin, Bremen and Hamburg) [71]. Buyers’ residence (federal state as well as town or city) was determined based on shipping addresses and postal codes. Numbers of inhabitants of German towns and cities (as of 01 June 2025) were retrieved from the *Gemeindeverzeichnis-Online* (List of Municipalities) [72]. Numbers of inhabitants of the German federal states (as of 31 December 2023) were retrieved from the Federal Statistical Office (Destatis) [73].

Towns and cities were categorized based on their number of inhabitants according to the Federal Institute for Research on Building, Urban Affairs and Spatial Development [74]:

- Rural municipalities: 1–4999 inhabitants
- Small towns: 5000–19,999 inhabitants
- Medium-sized cities: 20,000–99,999 inhabitants
- Large cities: ≥ 100,000 inhabitants

### Statistical methods

Descriptive statistical techniques were used to summarize the data. Categorical variables are reported as absolute and relative frequencies. Quantitative variables were tested for normal distribution with the Shapiro–Wilk test and by inspection of histograms and Q–Q plots. Due to skewed distributions, quantitative variables are depicted as medians with interquartile ranges (IQRs) or 95% confidence intervals (CIs). Means with standard deviations (SDs) are only reported where this is necessary for direct comparisons with results from other studies. The Mann–Whitney *U* test or Kruskal–Wallis test was used to investigate potential differences between two groups or ≥ three groups of quantitative variables, respectively. The log-rank test was applied to compare “survival” distributions (Kaplan–Meier curves), and Cox proportional hazards regression analyses (both univariable and multivariable models) were used to calculate hazard ratios (HRs) with 95% CIs [75]. The multivariable Cox regression was performed using the “Enter” method (i.e., all covariates were entered simultaneously into the model). Variables were entered into the model with a significance level of *p* ≤ 0.05 and removed if *p* ≥ 0.10. The maximum number of iterations was set to 20. The proportional hazards assumption was checked by visual assessment of Kaplan–Meier curves and log-minus-log plots (curves roughly parallel and not crossing). Of note, in this study, HRs > 1 were favorable from the seller’s point of view as they denoted an increased chance of cards being sold during the study period (compared to the respective reference category).

Fundamentally, *p*-values (two-sided) < 0.05 were considered statistically significant. For pairwise comparisons in Kruskal–Wallis tests, log-rank tests and univariable Cox regressions, the Bonferroni correction was applied to adjust for multiple testing (adjusted *p*-value (*p*_adj_) = *p*-value × number of pairwise comparisons). In the Kruskal–Wallis tests and the log-rank tests, three, six and 36 pairwise comparisons were conducted for the variables rarity (containing three values; 3 × (3–1)/2 = 3), residence (containing four values; 4 × (4–1)/2 = 6) and Pokémon trading card sets (containing nine values; 9 × (9–1)/2 = 36), respectively. In univariable Cox regressions, two and eight pairwise comparisons were conducted for the variables rarity and Pokémon trading card sets, respectively (one value always served as reference category against which the HRs of the other values were calculated). In the multivariable Cox regression, no adjustments for multiple testing were made [75].

All statistical analyses were performed with Microsoft Excel 2019 (Redmond, Washington, USA) and IBM SPSS Statistics for Windows, version 29 (Armonk, New York, USA). Choropleth maps were created with Datawrapper (Berlin, Germany) [76] and annotated with Microsoft PowerPoint 2016 (Redmond, Washington, USA).

## Results

### Trading card characteristics

Of 300 Pokémon trading cards offered on eBay during the study period, the most frequent card type were Pokémon (character) cards (91.0% (273/300)), followed by Trainer (8.0% (24/300)) and Energy cards (1.0% (3/300); Table 1). The three most prevalent card sets were the Base Set (41.7% (125/300)), Team Rocket (20.3% (61/300)), and Jungle set (11.3% (34/300)). With respect to rarity, Common cards were most frequent (48.0% (144/300)), followed by Uncommon (34.3% (103/300)) and Rare cards (17.3% (52/300)). For one card (i.e., a Black Star Promo card), rarity was considered not applicable. 9.3% (28/300) of the trading cards were holofoils, whereas the remaining 90.7% (272/300) were matte. The majority of cards were in German (71.0% (213/300)) or English language (28.3% (85/300)); only one card each was in Japanese and Italian. Only a minority of cards were from the first edition (2% (6/300)). The most frequent card conditions were Excellent (49.0% (147/300)) and Near Mint or Better (47.3% (142/300)). One card was a misprint.

**Table 1:** Characteristics of Pokémon trading cards offered (n = 300) and sold (n = 220) on the e-commerce platform eBay during the study period. The total revenue achieved was 923.60 €.

Variable	No. of cards offered	% of all cards offered	No. of cards sold	% of all cards sold	Cumulated revenue (€)	% of total revenue
**Card type**						
Pokémon	273	91.0	206	93.6	849.75	92.0
Trainer	24	8.0	12	5.5	59.85	6.5
Energy	3	1.0	2	0.9	14.00	1.5
**Card set**						
Base Set	125	41.7	86	39.1	436.16	47.2
Team Rocket	61	20.3	54	24.5	200.45	21.7
Jungle	34	11.3	24	10.9	102.20	11.1
Fossil	33	11.0	24	10.9	59.75	6.5
Base Set 2	21	7.0	12	5.5	32.45	3.5
Neo Genesis	14	4.7	8	3.6	30.70	3.3
Gym Challenge	10	3.3	10	4.5	48.89	5.3
Gym Heroes	1	0.3	1	0.5	6.50	0.7
Black Star Promo	1	0.3	1	0.5	6.50	0.7
**Rarity**						
Common	144	48.0	96	43.6	174.07	18.8
Uncommon	103	34.3	81	36.8	199.58	21.6
Rare	52	17.3	42	19.1	543.45	58.8
Not applicable	1	0.3	1	0.5	6.50	0.7
**Holographic effect**						
Non-holofoil (matte)	272	90.7	196	89.1	510.35	55.3
Holofoil	28	9.3	24	10.9	413.25	44.7
**Language**						
German	213	71.0	155	70.5	707.86	76.6
English	85	28.3	63	28.6	207.74	22.5
Japanese	1	0.3	1	0.5	6.50	0.7
Italian	1	0.3	1	0.5	1.50	0.2
**Edition**						
Unlimited edition	294	98.0	217	98.6	902.25	97.7
First edition	6	2.0	3	1.4	21.35	2.3
**Card condition**						
Near Mint or Better	142	47.3	105	47.7	334.56	36.2
Excellent	147	49.0	108	49.1	552.14	59.8
Very Good	11	3.7	7	3.2	36.90	4.0
Poor	0	0	–	–	–	–
**Print quality**						
Correctly printed	299	99.7	220	100	923.60	100
Misprint (error card)	1	0.3	0	0	0	0

Abbreviations: e-commerce, electronic commerce; no., number.

### Sales statistics

Of 300 Pokémon trading cards offered on eBay, 220 were sold during the study period (73.3%), generating a total revenue of 923.60 €. The characteristics of sold cards, along with cumulated revenues and proportions of the total revenue, are displayed in Table 1. The median revenue per trading card was 1.95 € (IQR 1.31–3.15 €, mean ± SD: 4.20 ± 10.38 €, minimum: 1.00 €, maximum: 145.00 €; S2 Table), with statistically significant differences between Rare, Uncommon, and Common cards (global *p* < 0.001; Table 2). In pairwise comparisons, Rare cards (median 9.48 € (IQR 3.99–13.96 €))) fetched significantly higher sales prices than Uncommon (median 1.95 € (IQR 1.45–2.98 €), *p*_adj_ < 0.001) and Common cards (median 1.50 € (IQR 1.14–2.31 €), *p*_adj_ < 0.001); Uncommon cards sold at significantly higher prices than Common cards (*p*_adj_ = 0.019). Remarkably, while Rare cards accounted for only 19.1% (42/220) of all cards sold, they contributed 58.8% (543.45 €/923.60 €) to the total revenue.

**Table 2:** Comparison of sales prices of Pokémon trading cards (n = 220) across different variables.

Variable	No. of cards sold (% of all cards sold)	Sales price (€)—median (IQR)	Global *p*-value
**Set**			
Base Set	86 (39.1)	1.98 (1.24–3.54)	0.572^b^
Team Rocket	54 (24.5)	2.10 (1.33–3.74)	
Fossil	24 (10.9)	1.70 (1.46–2.15)	
Jungle	24 (10.9)	1.88 (1.15–5.26)	
Base Set 2	12 (5.5)	1.50 (1.36–2.38)	
Gym Challenge	10 (4.5)	2.08 (1.60–3.04)	
Neo Genesis	8 (3.6)	2.68 (2.48–3.13)	
Black Star Promo	1 (0.5)	6.50^a^	
Gym Heroes	1 (0.5)	6.50^a^	
**Rarity**			
Common	96 (43.6)	1.50 (1.14–2.31)	**<0.001** ^**b**^
Uncommon	81 (36.8)	1.95 (1.45–2.98)	
Rare	42 (19.1)	9.48 (3.99–13.96)	
Not applicable^c^	1 (0.5)	6.50^a^	n.a.
**Holographic effect**			
Non-holofoil (matte)	196 (89.1)	1.85 (1.21–2.95)	**<0.001** ^**d**^
Holofoil	24 (10.9)	13.03 (7.24–15.00)	
**Language**			
German	155 (70.5)	2.15 (1.35–3.65)	0.076^d^
Non-German	65 (29.5)	1.80 (1.20–2.95)	
**Edition**			
Unlimited edition	217 (98.6)	1.95 (1.29–3.15)	0.060^d^
First edition	3 (1.4)	5.00 (2.85–n.e.)	
**Card condition**			
Near Mint or Better	105 (47.7)	2.00 (1.20–3.05)	0.411^b^
Excellent	108 (49.1)	1.95 (1.38–3.61)	
Very Good	7 (3.2)	5.00 (1.15–7.95)	

^a^Only the exact numerical value is indicated as only one card was sold for this set.

^b^Kruskal–Wallis test. Significant global *p*-values are highlighted in bold. Where applicable, significant adjusted *p*-values for pairwise comparisons are depicted in the text.

^c^Values from this category were excluded from inferential statistics.

^d^Mann–Whitney *U* test. Significant *p*-values are highlighted in bold.

Abbreviations: IQR, interquartile range; n.e., not estimable; no., number.

Sales prices did not differ significantly across Pokémon trading card sets (global *p* = 0.572), and the individual contributions of the different sets to the total revenue largely reflected their proportions of all cards sold.

Holofoil cards achieved significantly higher sales prices compared to matte cards (median 13.03 € (IQR 7.24–15.00 €) vs. median 1.85 € (IQR 1.21–2.95 €), *p* < 0.001). Of note, while holofoils accounted for only 10.9% (24/220) of all cards sold, they contributed 44.7% (413.25 €/923.60 €) to the total revenue.

We did not detect statistically significant differences for sales prices between trading cards in German language and non-German cards (median 2.15 € (IQR 1.35–3.65 €) vs. median 1.80 € (IQR 1.20–2.95 €), *p* = 0.076), nor between first-edition and unlimited-edition cards (median 5.00 € (IQR 2.85 €–n.e.) vs. median 1.95 € (IQR 1.29–3.15 €), *p* = 0.060), nor between cards of different conditions (Near Mint or Better: median 2.00 € (IQR 1.20–3.05 €), Excellent: median 1.95 € (1.38–3.61 €), Very Good: median 5.00 € (IQR 1.15–7.95 €), global *p* = 0.411).

### Event history analyses

For event history analyses, “survival” times of Pokémon trading cards were defined as the time between offer and sale for sold cards, or as the time between offer and the end of the study period for unsold cards (reflecting censored data).

**Fig 1:**
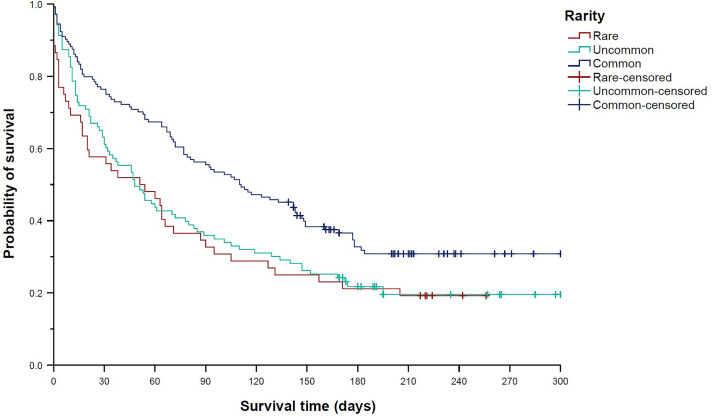
Kaplan–Meier curves showing “survival” distributions of Pokémon trading cards offered on eBay (n = 300). Survival time was defined as the time between offer and sale for sold cards, or as the time between offer and the end of the study period for unsold cards (reflecting censored data). Comparison of cards of different grades of rarity.

Survival distributions differed significantly across the three rarity categories (global *p* = 0.005; Fig 1). Pairwise comparisons demonstrated a significantly longer survival time (reflecting slower sales kinetics) of Common cards (median 110 days (95% CI 74–146 days) compared to Rare (median 51 days (95% CI 13–89 days), *p*_adj_ = 0.020) and Uncommon cards (median 48 days (95% CI 30–66 days), *p*_adj_ = 0.018). Correspondingly, Rare and Uncommon cards had 64% and 50% higher chances of being sold during the study period compared to Common cards (HR 1.64 (95% CI 1.14–2.36), *p*_adj_ = 0.015; and HR 1.50 (95% CI 1.11–2.01), *p*_adj_ = 0.015, respectively).

**Fig 2:**
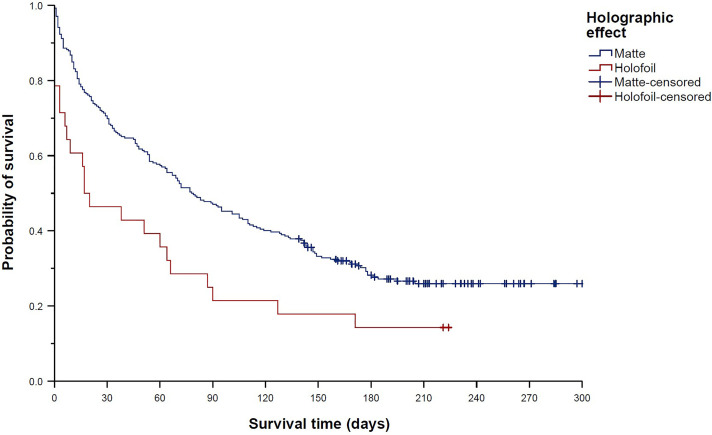
Kaplan–Meier curves showing “survival” distributions of Pokémon trading cards offered on eBay (n = 300). Survival time was defined as the time between offer and sale for sold cards, or as the time between offer and the end of the study period for unsold cards (reflecting censored data). Comparison of holofoil and matte cards.

**Fig 3:**
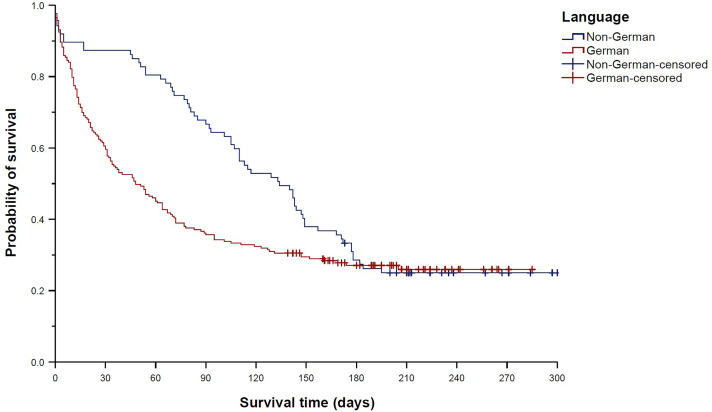
Kaplan–Meier curves showing “survival” distributions of Pokémon trading cards offered on eBay (n = 300). Survival time was defined as the time between offer and sale for sold cards, or as the time between offer and the end of the study period for unsold cards (reflecting censored data). Comparison of cards in German language and non-German cards.

The survival time was significantly shorter (reflecting faster sales kinetics) for holofoil cards than for matte cards (median 17 days (95% CI 0–46 days) vs. median 78 days (95% CI 58–98 days), *p* = 0.006; Fig 2). Accordingly, holofoils displayed a 79% higher chance of being sold than matte cards (HR 1.79 (95% CI 1.17–2.74), *p* = 0.007). The survival time was also significantly shorter for cards in German language than for non-German cards (median 48 days (95% CI 31–65 days) vs. median 134 days (95% CI 107–161 days), *p* = 0.021; Fig 3); hence, cards in German language showed a 40% increased chance of being sold compared to non-German cards (HR 1.40 (95% CI 1.05–1.88), *p* = 0.022).

**Fig 4:**
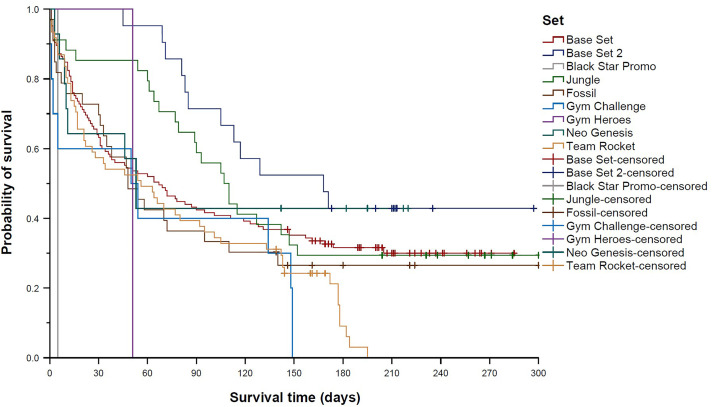
Kaplan–Meier curves showing “survival” distributions of Pokémon trading cards offered on eBay (n = 300). Survival time was defined as the time between offer and sale for sold cards, or as the time between offer and the end of the study period for unsold cards (reflecting censored data). Comparison of cards from nine different sets.

**Fig 5:**
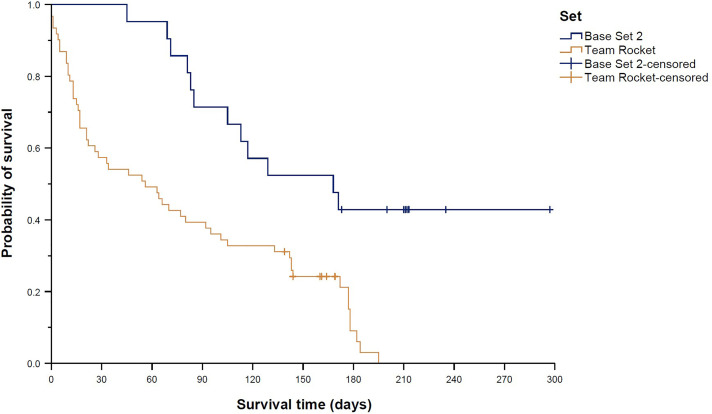
Kaplan–Meier curves showing “survival” distributions of Pokémon trading cards offered on eBay (n = 300). Survival time was defined as the time between offer and sale for sold cards, or as the time between offer and the end of the study period for unsold cards (reflecting censored data). Pairwise log-rank tests with adjustments for multiple testing revealed two significantly different survival distributions: Base Set 2 vs. Team Rocket (shown here), and Base Set 2 vs. Black Star Promo (shown in Fig 6).

**Fig 6:**
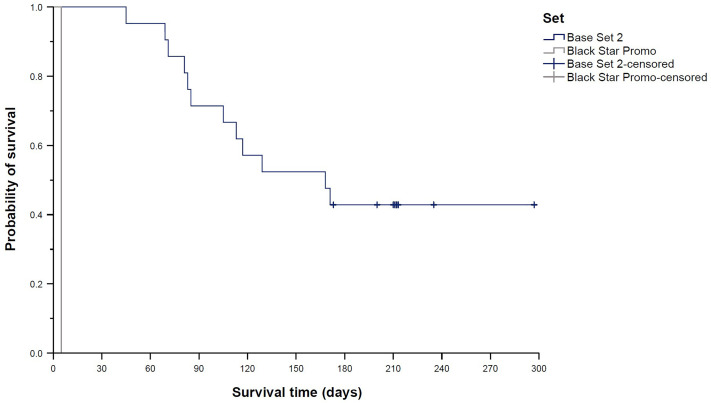
Kaplan–Meier curves showing “survival” distributions of Pokémon trading cards offered on eBay (n = 300). Survival time was defined as the time between offer and sale for sold cards, or as the time between offer and the end of the study period for unsold cards (reflecting censored data). Pairwise log-rank tests with adjustments for multiple testing revealed two significantly different survival distributions: Base Set 2 vs. Team Rocket (shown in Fig 5), and Base Set 2 vs. Black Star Promo (shown here). Of note, only one Black Star Promo card was contained in the sample, suggesting that this result should be interpreted with caution.

**Fig 7:**
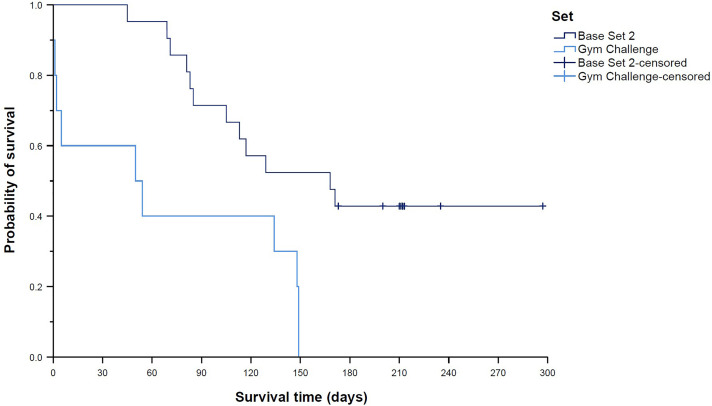
Kaplan–Meier curves showing “survival” distributions of Pokémon trading cards offered on eBay (n = 300). Survival time was defined as the time between offer and sale for sold cards, or as the time between offer and the end of the study period for unsold cards (reflecting censored data). Cox regression analysis additionally indicated an increased hazard ratio (i.e., a higher chance of being sold during the study period) for Gym Challenge cards compared to Base Set 2 cards. Details are provided in the text.

Survival distributions differed significantly across the nine trading card sets contained in the sample (global *p* = 0.004; Fig 4). Pairwise comparisons revealed a significantly longer survival time of Base Set 2 cards (median 168 days (95% CI 87–249 days) compared to Team Rocket cards (median 56 days (95% CI 20–92 days), *p*_adj_ = 0.007; Fig 5) and the Black Star Promo card (survival time 5 days (95% CI n.e.), *p*_adj_ < 0.001; Fig 6). In line with this finding, Team Rocket cards had a 163% higher chance of being sold than Base Set 2 cards (HR 2.63 (95% CI 1.41–4.93), *p*_adj_ = 0.020). Cox regression analysis also indicated an increased HR for the Black Star Promo card (14.40 (95% CI 1.83–113.34)), but the adjusted *p*-value was not significant (*p*_adj_ = 0.090). Instead, Cox regression analysis showed a 229% higher chance of being sold for Gym Challenge cards compared to Base Set 2 cards (HR 3.29 (95% CI 1.42–7.63), *p*_adj_ = 0.044; Fig 7); however, the median survival times of those two sets were not significantly different according to the log-rank test (median 50 days (95% CI 0–126 days) vs. median 168 days (95% CI 87–249 days), *p*_adj_ = 0.087).

Survival distributions did not differ significantly between first-edition and unlimited-edition cards (median survival time 95 days (95% CI n.e.) vs. median survival time 71 days (95% CI 55–87 days), *p* = 0.206), nor between cards of different conditions (Near Mint or Better: median survival time 77 days (95% CI 44–110 days), Excellent: median survival time 67 days (95% CI 51–83 days), Very Good: median survival time 127 days (95% CI 18–236 days), global *p* = 0.565).

In addition to the univariable Cox regression analyses reported above, a multivariable Cox regression was performed to examine the effects of set, rarity, language, holographic effect, edition and card condition on survival time in a unified, comprehensive model. The results of the univariable analyses and of the multivariable model are juxtaposed in Table 3. While the multivariable model overall corroborated the findings of the univariable analyses with respect to set (with the exception that the Black Star Promo card was excluded from the multivariable model by SPSS), language and holographic effect, differences were observed with regard to rarity, edition and card condition. In the multivariable Cox regression model, Uncommon cards featured a 68% higher chance of being sold during the study period than Common cards (HR 1.68 (95% CI 1.23–2.29), *p* = 0.001), but there was no significant difference between Rare and Common cards (in contrast to the univariable analysis). The multivariable model showed a 304% higher chance of being sold for unlimited-edition compared to first-edition cards (HR 4.04 (95% CI 1.26–13.00), *p* = 0.019), while there was no difference in the univariable analysis. However, this apparent edition effect was based on a very small subgroup (with only n = 6 first-edition cards contained in the sample) and is therefore likely unstable; caution should be exercised when interpreting this result. Finally, cards in Excellent and cards in Near Mint or Better condition displayed 212% and 260% higher chances of being sold compared to cards in Very Good condition (HR 3.12 (95% CI 1.31–7.43), *p* = 0.010; and HR 3.60 (95% CI 1.46–8.92), *p* = 0.006, respectively), while there were no significant differences in the corresponding univariable analysis.

**Table 3:** Results of univariable and multivariable Cox regression analyses.

Variable	Univariable Cox regressions		Multivariable Cox regression	
	HR (95 % CI)	Adjusted *p*-value	HR (95 % CI)	Global *p*-value
**Set**				
Base Set 2	reference		reference	**< 0.001**
Base Set	1.78 (0.97–3.26)	0.494	0.75 (0.36–1.58)	
Team Rocket	**2.63 (1.41–4.93)**	**0.020**	**1.98 (1.01–3.91)**	
Fossil	2.14 (1.07–4.29)	0.250	1.11 (0.50–2.43)	
Jungle	1.50 (0.75–2.99)	1.000	1.24 (0.61–2.52)	
Gym Challenge	**3.29 (1.42–7.63)**	**0.044**	**3.49 (1.49–8.19)**	
Neo Genesis	1.47 (0.60–3.60)	1.000	0.60 (0.22–1.65)	
Black Star Promo	14.40 (1.83–113.34)	0.090	excluded from multivariable Cox regression	
Gym Heroes	3.67 (0.47–28.33)	1.000	1.16 (0.14–9.61)	
**Rarity**				
Common	reference		reference	**0.005**
Uncommon	**1.50 (1.11–2.01)**	**0.015**	**1.68 (1.23–2.29)**	
Rare	**1.64 (1.14–2.36)**	**0.015**	1.29 (0.75–2.21)	
**Holographic effect**				
Non-holofoil (matte)	reference		reference	**< 0.001**
Holofoil	**1.79 (1.17–2.74)**	**0.007**	**3.18 (1.62–6.25)**	
**Language**				
Non-German	reference		reference	**< 0.001**
German	**1.40 (1.05–1.88)**	**0.022**	**2.35 (1.52–3.64)**	
**Edition**				
First edition	reference		reference	**0.019**
Unlimited edition	2.05 (0.66–6.40)	0.218	**4.04 (1.26–13.00)**	
**Card condition**				
Very Good	reference		reference	**0.021**
Excellent	1.51 (0.70–3.24)	0.582	**3.12 (1.31–7.43)**	
Near Mint or Better	1.46 (0.68–3.15)	0.658	**3.60 (1.46–8.92)**	

Abbreviations: CI, confidence interval; HR, hazard ratio.

### Buyer demographics

Pokémon trading card buyers were predominantly male (91.8% (202/220)), but on average female buyers spent more money per trading card than male buyers (median 3.05 € (IQR 1.97–8.98 €, mean ± SD: 5.35 ± 4.63 €) vs. median 1.95 € (IQR 1.34–3.15 €, mean ± SD: 4.12 ± 10.75 €), *p* = 0.022). Latencies between offer and sale of Pokémon trading cards, by contrast, did not differ between female and male buyers (median 54 days (IQR 12–97 days) vs. median 36 days (IQR 11–88 days), *p* = 0.490).

Pokémon trading card buyers mostly lived in large cities (40.0% (88/220)), followed by small towns (34.5% (76/220); Table 4). Interestingly, while buyers residing in medium-sized cities only purchased 19.5% (43/220) of all cards, they contributed 32.1% (296.63 €/923.60 €) to the total revenue. Latencies between offer and sale of Pokémon trading cards differed significantly across residence (global *p* = 0.030), with trends to shorter latencies for inhabitants of rural municipalities (median 14 days (IQR 2–81 days), *p* = 0.014, *p*_adj_ = 0.082) and medium-sized cities (median 31 days (IQR 11–83 days), *p* = 0.041, *p*_adj_ = 0.248) compared to inhabitants of large cities (median 47 days (IQR 13–143 days)).

**Table 4:** Comparison of buyer demographics with respect to cumulated revenues, sales prices and latencies between offer and sale of Pokémon trading cards. In total, 220 trading cards were sold during the study period, generating a total revenue of 923.60 €.

Variable	No. of cards sold	% of all cards sold	Cumulated revenue (€)	% of total revenue	Sales price (€)—median (IQR)	Global *p*-value	Latency (days)—median (IQR)	Global *p*-value
**Gender**								
Male	202	91.8	831.52	90.0	1.95 (1.34–3.15)	**0.022** ^**c**^	36 (11–88)	0.490^c^
Female	17	7.7	90.93	9.8	3.05 (1.97–8.98)		54 (12–97)	
Not determinable^a^	1	0.5	1.15	0.1	1.15^b^	n.a.	89^b^	n.a.
**Residence**								
Large cities	88	40.0	304.70	33.0	1.83 (1.20–3.61)	0.478^d^	47 (13–143)	**0.030** ^**d**^
Medium-sized cities	43	19.5	296.63	32.1	1.85 (1.15–3.95)		31 (11–83)	
Small towns	76	34.5	243.42	26.4	2.00 (1.50–3.05)		48 (12–69)	
Rural municipalities	13	5.9	78.85	8.5	2.95 (1.20–12.55)		14 (2–81)	

^a^Values from this category were excluded from inferential statistics.

^b^Only the exact numerical value is indicated as only one card was sold for this category.

^c^Mann–Whitney *U* test. Significant *p*-values are highlighted in bold.

^d^Kruskal–Wallis test. Significant global *p*-values are highlighted in bold. Where applicable, significant *p*-values for pairwise comparisons are depicted in the text.

Abbreviations: IQR, interquartile range; n.a., not applicable; no., number.

**Fig 8:**
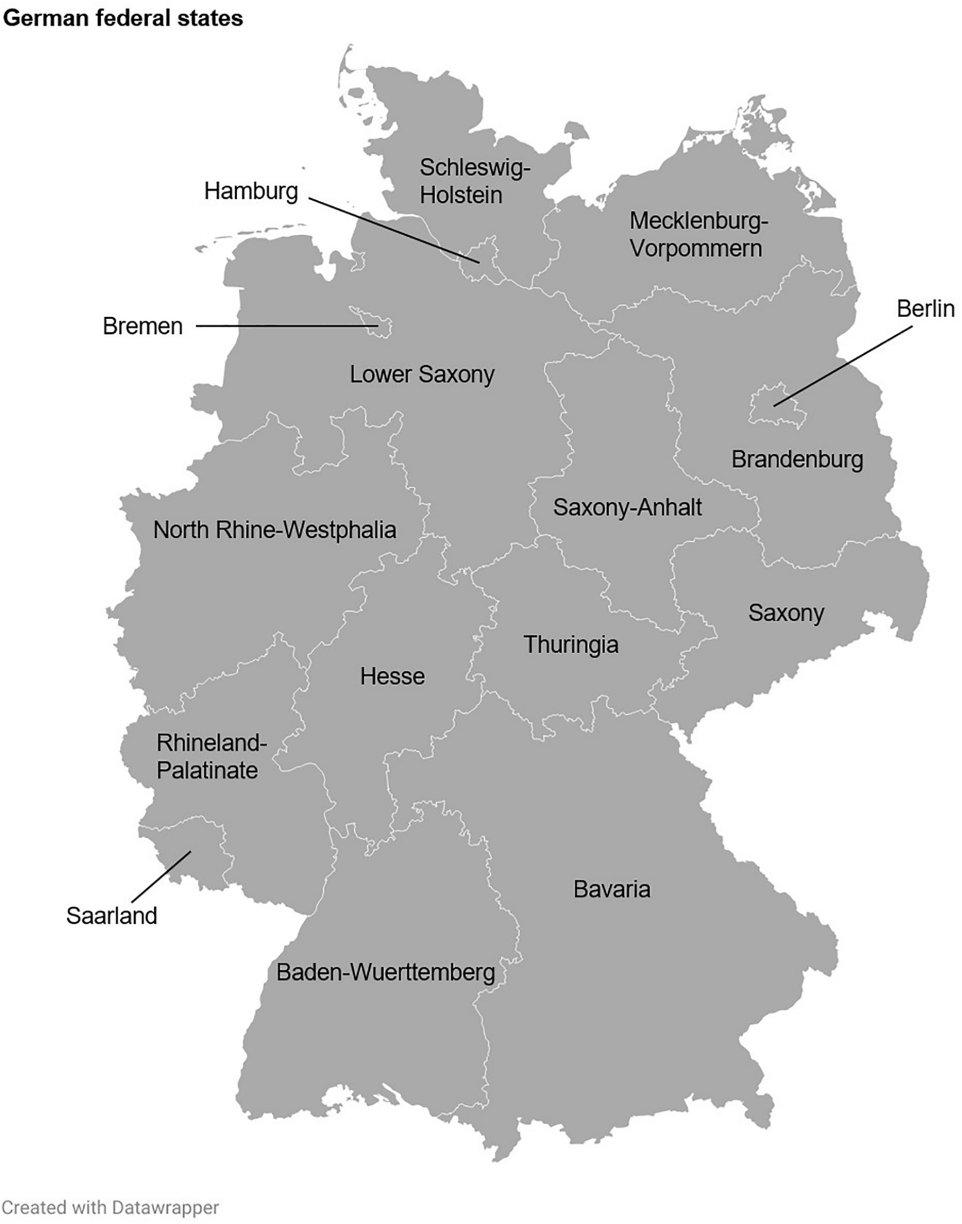
Sales distributions of Pokémon trading cards and associated revenues across the Federal Republic of Germany. Geographic location of the 16 German federal states.

**Fig 9:**
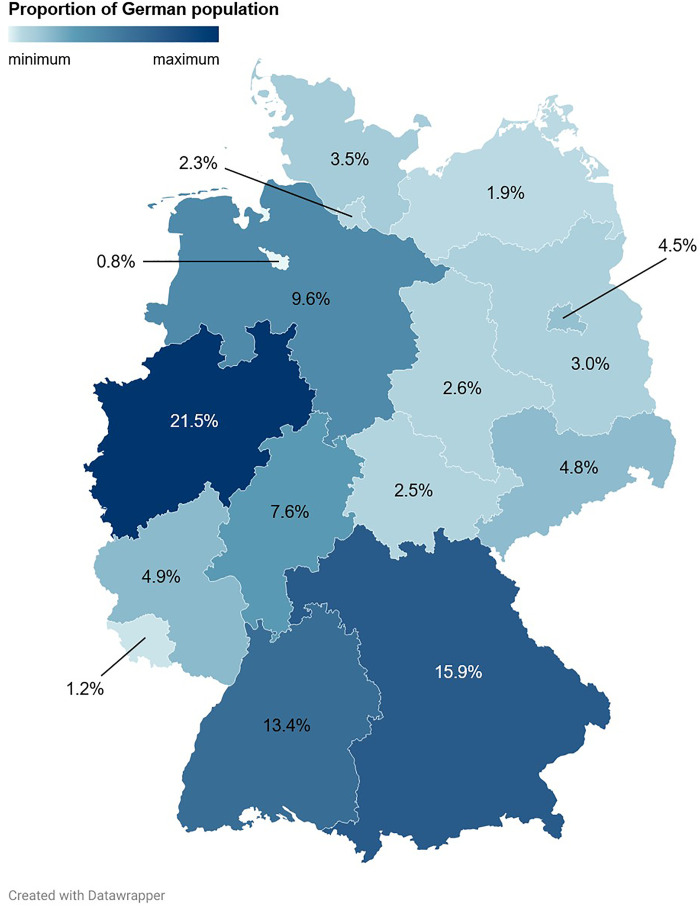
Sales distributions of Pokémon trading cards and associated revenues across the Federal Republic of Germany. Shown are the proportions of the German population per federal state.

**Fig 10:**
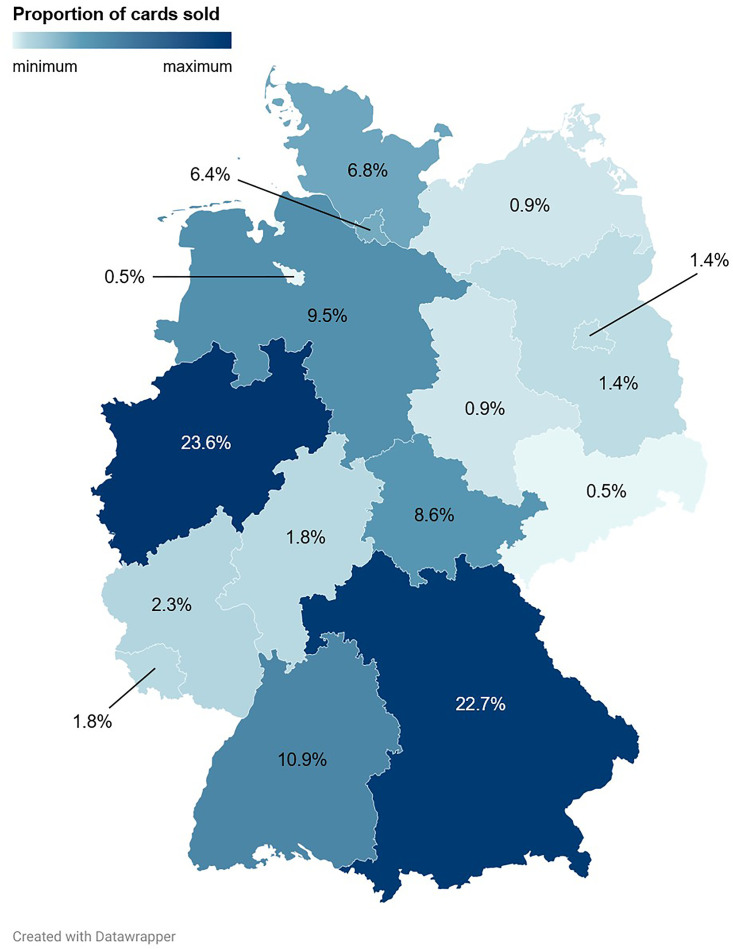
Sales distributions of Pokémon trading cards and associated revenues across the Federal Republic of Germany. During the study period, 220 cards were sold, generating a total revenue of 923.60 €. Shown are the proportions of Pokémon trading cards sold per federal state.

The three German federal states in which the most trading cards were sold were North Rhine-Westphalia (23.6% (52/220)), Bavaria (22.7% (50/220)), and Baden-Wuerttemberg (10.9% (24/220); Fig 8, Fig 9, Fig 10 and S3 Table). North Rhine-Westphalia and Bavaria also ranked among the top three German federal states that contributed the most to the total revenue, accounting for 19.5% (179.70 €/923.60 €) and 16.3% (150.17 €/923.60 €) of the total revenue, respectively (Fig 12). Interestingly, the second rank in this analysis was occupied by Brandenburg, which contributed 17.5% (161.90 €/923.60 €) to the total revenue. This can be explained by the most expensive card of the sample, a Glurak (English name: Charizard) 4/102 holofoil from the Base Set, which sold for 145.00 € to a buyer from Brandenburg, and which alone contributed 15.7% to the total revenue.

**Fig 11:**
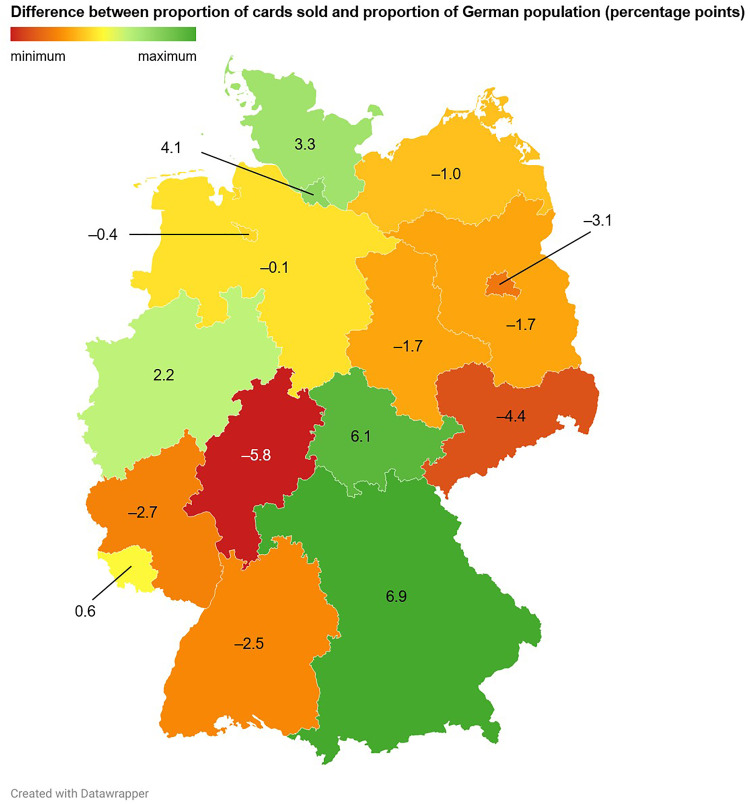
Sales distributions of Pokémon trading cards and associated revenues across the Federal Republic of Germany. During the study period, 220 cards were sold, generating a total revenue of 923.60 €. Shown are the differences between the proportions of all cards sold and the proportions of the German population living in the respective federal states, expressed in percentage points.

Bavaria, Thuringia, Hamburg and Schleswig-Holstein displayed favorable Pokémon trading card sales potentials (defined as the differences between the proportions of all cards sold and the proportions of the German population living in the respective federal states, expressed in percentage points (PP); Bavaria: 6.9 PP, Thuringia: 6.1 PP, Hamburg: 4.1 PP, Schleswig-Holstein: 3.3 PP), while Hesse (–5.8 PP), Saxony (–4.4 PP), Berlin (–3.1 PP) and Rhineland-Palatinate (–2.7 PP) exhibited unfavorable sales potentials (Fig 11 and S3 Table).

On the other hand, Brandenburg, Thuringia and Hamburg showed favorable Pokémon trading card revenue potentials (defined as the differences between the proportions of the total revenue and the proportions of the German population living in the respective federal states, expressed in percentage points (PP); Brandenburg: 14.5 PP, Thuringia: 5.0 PP, Hamburg: 3.2 PP), whereas Hesse (–6.1 PP), Baden-Wuerttemberg (–4.9 PP), Saxony (–4.5 PP) and Berlin (–2.7 PP) demonstrated unfavorable revenue potentials (Fig 13 and S3 Table).

Taken together, Thuringia and Hamburg consistently displayed positive economic potentials for Pokémon trading card sellers, while Hesse, Saxony and Berlin were characterized by negative economic prospects. Economic prospects were potentially favorable for Bavaria (comparatively high number of cards sold but cumulated revenue only average) and Brandenburg (comparatively high cumulated revenue but rather low number of cards sold). Of note, economic prospects for Brandenburg should be interpreted with caution since a single card with an outlier sales price massively impacted on the cumulated revenue.

### Artificial intelligence-supported sales price estimation

The AI tool Perplexity was able to calculate market price ranges for 299 out of 300 Pokémon trading cards (99.7%). For one Pokémon trading card (a Flareon 19/64 card from the Jungle set), Perplexity was unable to determine a market price range. After manual reevaluation on eBay, price offers (posted prices) within (65.2% (195/299)), below (21.1% (63/299)) or above Perplexity price ranges (13.7% (41/299)) were selected (Fig 14). Price offers for Pokémon trading cards below or above Perplexity price ranges were chosen if there was comparatively strong or weak competition on eBay (i.e., high or low number of similar cards on offer), respectively. Taken together, the AI price range suggestions were overridden by the human seller in 34.8% (104/299) of cases.

Comparison of sales prices for 219 out of 220 Pokémon trading cards sold during the study period demonstrated that the initial Perplexity price ranges could be achieved in 60.7% (133/219) of cases when Perplexity was used as a decision support tool but the human seller remained in charge of the final decision about posted prices (Fig 15). Hence, the 60.7% represent an aggregate measure composed of cases in which the human seller trusted Perplexity and cases in which Perplexity was overridden. In 29.7% (65/219) and 9.6% (21/219) of cases, Pokémon trading cards sold below or above their initial Perplexity price ranges.

Of the 195 cards for which the AI price range suggestions had been adopted for posted prices in the first place, 141 cards were sold during the study period (72.3%). When only those cards were considered, Perplexity price ranges could be achieved in 87.2% (123/141) of cases (Fig 16). In the remaining 12.8% (18/141) of cases, Pokémon trading cards sold below their Perplexity price ranges.

### ABC analysis

To gain insight into how sales activities for Pokémon trading cards could be prioritized, and ABC analysis was conducted. The ABC analysis is a prioritization technique with a wide range of possible applications for segmenting items based on their contribution to total value, dividing them into three categories: “A” – the most valuable items, “B” – items of intermediate value, and “C” – the least valuable items [77–79]. For the purpose of this study, all 220 sold trading cards were sorted in order of descending sales prices (S1 Dataset). “A” cards were defined as cards with a cumulated revenue of 80% of the total revenue (100% = 923.60 €), “B” cards contributed the next 15% to the total revenue (i.e., from 80% to 95%), and “C” cards accounted for the remaining 5% of the total revenue (i.e., from 95% to 100%) [77–79].

The 93 most valuable cards (42.3% of all cards sold) were “A” cards, the following 84 cards (38.2%) were “B” cards, and the last 43 cards (19.5%) were “C” cards. The Lorenz curve in Fig 17 depicts this uneven distribution graphically. Of note, 43.0% (40/93) and 24.7% (23/93) of “A” cards were Rare and holofoil cards, respectively. “B” cards, by contrast, only contained 2.2% (2/84) Rare and 1.1% (1/84) holofoil cards. Lastly, no Rare nor holofoil cards were present among “C” cards.

**Fig 12:**
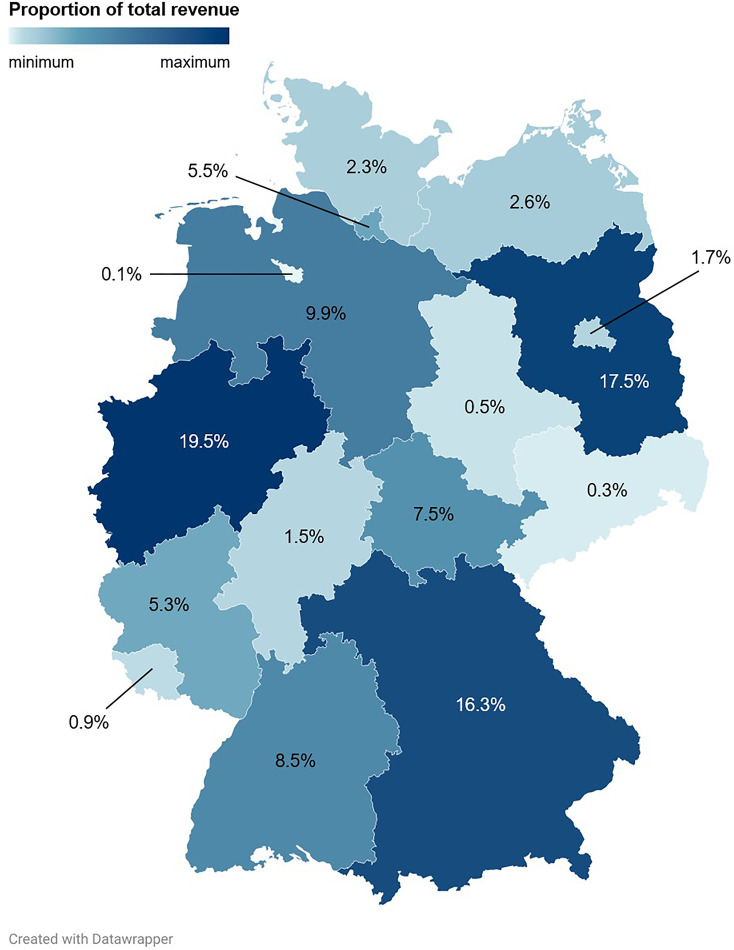
Sales distributions of Pokémon trading cards and associated revenues across the Federal Republic of Germany. During the study period, 220 cards were sold, generating a total revenue of 923.60 €. Shown are the proportions of the total revenue per federal state.

**Fig 13:**
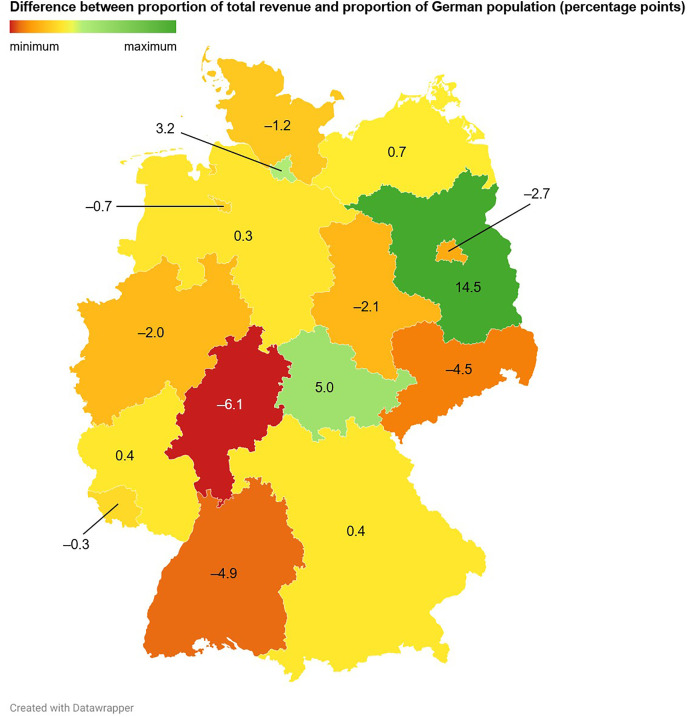
Sales distributions of Pokémon trading cards and associated revenues across the Federal Republic of Germany. During the study period, 220 cards were sold, generating a total revenue of 923.60 €. Shown are the differences between the proportions of the total revenue and the proportions of the German population living in the respective federal states, expressed in percentage points.

**Fig 14:**
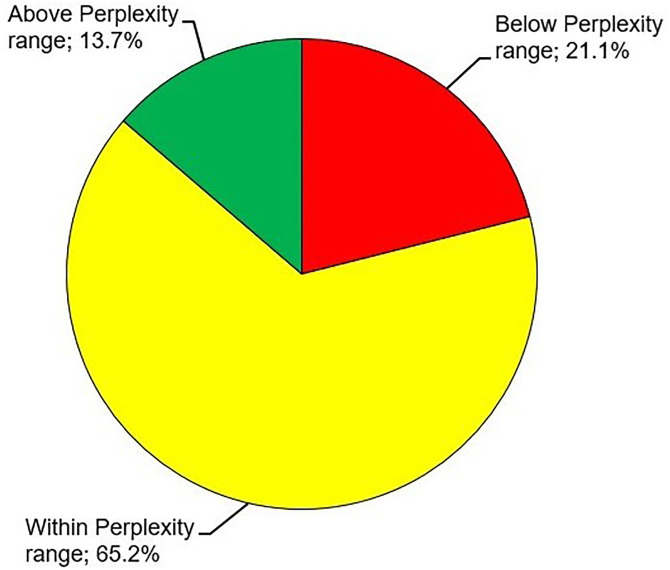
Artificial intelligence (AI)-supported sales price estimation of Pokémon trading cards. The AI application Perplexity was able to calculate market price ranges for 299 out of 300 Pokémon trading cards. For one card (a Flareon 19/64 from the Jungle set), Perplexity was unable to determine a market price range. After manual reevaluation on the e-commerce platform eBay, price offers (posted prices) within, below or above Perplexity price ranges were selected at the indicated proportions.

**Fig 15:**
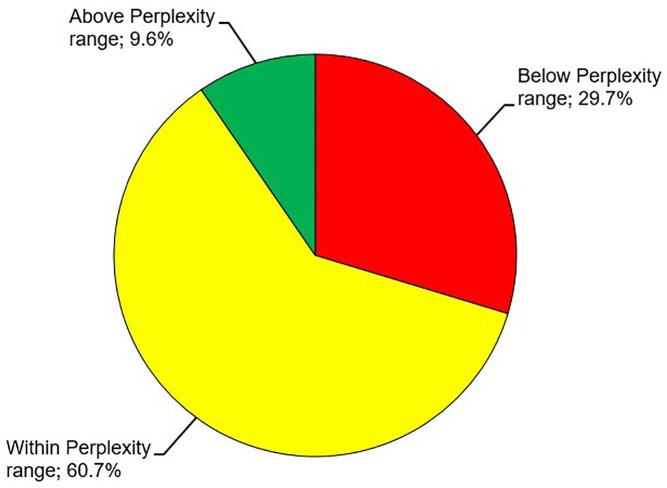
Artificial intelligence (AI)-supported sales price estimation of Pokémon trading cards. Sales prices for 219 out of 220 Pokémon trading cards sold during the study period were compared with their initial Perplexity price ranges. Proportions of sales prices that lay within, below or above Perplexity price ranges are shown.

**Fig 16:**
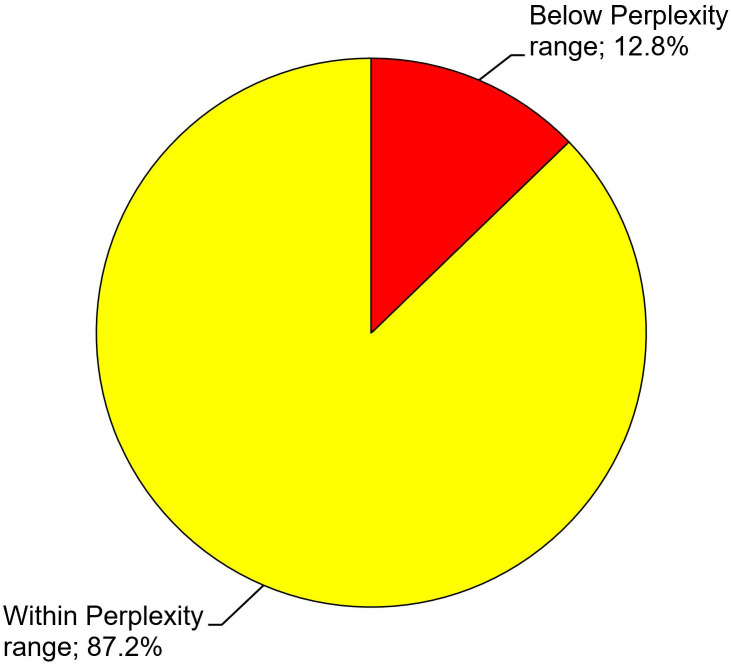
Artificial intelligence (AI)-supported sales price estimation of Pokémon trading cards. Sales prices for 141 out of 195 Pokémon trading cards for which the AI price range suggestions had been adopted for posted prices in the first place were compared with their Perplexity price ranges. Proportions of sales prices that lay within or below their Perplexity price ranges are displayed.

**Fig 17:**
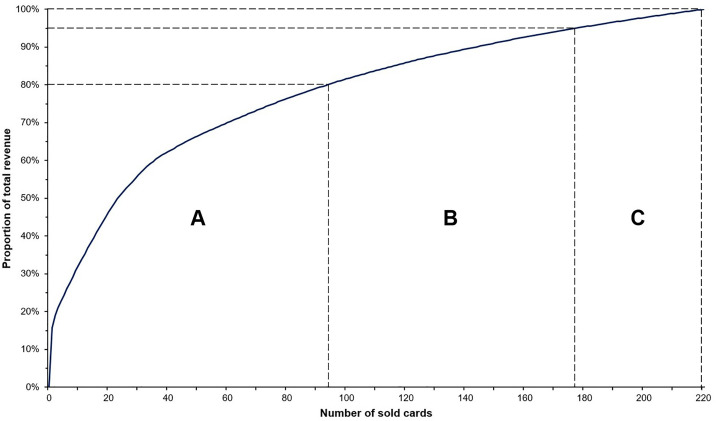
Lorenz curve as graphical representation of ABC analysis. All Pokémon trading cards sold during the study period (n = 220) were sorted in order of descending sales prices and plotted against their cumulative revenues (shown as proportions of the total revenue (100% = 923.60 €)). The initial sharp increase of the curve reflects the most expensive trading card of the sample, a Glurak (English name: Charizard) 4/102 holofoil from the Base Set, which sold for 145.00 € and alone contributed 15.7% to the total revenue. “A” cards were defined as cards with a cumulated revenue of 80% of the total revenue; “B” cards contributed the next 15% to the total revenue (i.e., from 80% to 95%), and “C” cards accounted for the remaining 5% of the total revenue (i.e., from 95% to 100%).

## Discussion

In this prospective one-year field study, we were able to sell 73.3% of a convenience sample of 300 Pokémon trading cards offered on the e-commerce platform eBay, generating a total revenue of 923.60 €. Similar to Magic the Gathering cards [26], we noticed a highly skewed sales price distribution of Pokémon trading cards. The mean of all cards sold in our investigation was 4.20 €, with a high SD of 10.38 €. In contrast to Weber [26] and David-Marshall et al. [9], but in line with Gergaud and Verardi [80], this prompted us to report medians with IQRs (e.g., for sales prices the median was 1.95 € with an IQR of 1.31–3.15 €), and to apply non-parametric inferential statistical methods for comparisons across groups. Of note, the sales price of the trading card with the highest value in our sample exceeded the sales price of the cheapest card by a factor of more than 100. For items with nearly identical material value (cards essentially consist of printed cardboard with or without a holofoil finish) [81], this highlights the decisive factor of rarity for the determination of market prices, a feature characteristic of collectibles [23,81–84]. A strength of our analysis is the study period of one year, which was considerably longer compared to the majority of previous investigations conducted on eBay, which had study periods between 1 day and 7 months [10,30–32,35–38,85,86]. In fact, we found only one eBay study with a longer study period than ours, namely the analysis by Hayne and colleagues, who investigated eBay auctions for 1968 Chevrolet Camaros over nearly 2 years [39]. Therefore, we suppose that potential seasonality effects—as detected by Bracker and Smith in their 31-day analysis of Glory Bear Beanie Babies auctions [38]—could effectively be controlled for in our study.

As expected, and similar to the results obtained by Gergaud and Verardi [80], we observed a declining price differential from Rare to Uncommon to Common cards (median sales price 9.48 € vs. 1.95 € vs. 1.50 €). In a 2009 market analysis, the average sales price for a Rare Pokémon trading card was US$7.56 [9] (corresponding to 9.74 € in 2025 when inflation [87] and the current exchange rate (US$1 = 0.8556 € on 15 August 2025) [88] are taken into account). Among conventional TCGs (average prices for Rare cards shown in brackets), this was only exceeded by Neopets (US$9.48), Naruto (US$9.40), and World of Warcraft (US$9.38) [9]. In 2009, the eBay TCG secondary market encompassed a total market volume of 605,370 card sales with a total revenue of US$938,385.61 [9]. Within this market, the Pokémon TCG occupied the third rank (34,474 cards sold (5.7%); cumulated revenue US$92,103.60 (9.8%)) after Magic the Gathering (345,986 cards sold (57.2%); US$438,979.20 (46.8%)) and Yu-Gi-Oh (79,682 cards sold (13.2%); US$221,170.34 (23.6%)) [9]. However, the average sales prices for Rare Magic the Gathering (US$4.32) and Yu-Gi-Oh cards (US$7.51) were lower compared to Rare Pokémon trading cards (US$7.56) [9].

In the present study, Rare and Uncommon cards sold significantly faster than Common cards, their median survival times being less than half that of Common cards (51 days and 48 days vs. 110 days). This finding was corroborated by the multivariable Cox regression analysis especially for Uncommon cards, which displayed a 68% increased chance of being sold during the study period compared to Common cards. This result may encourage sellers who are evaluating the economic potential of Uncommon cards. Of interest, for other TCGs like Magic the Gathering, finance speculators have even discussed investing in relatively cheap cards as “penny stocks”, with 100–400% gains over the course of 1–2 years [26,89]. It should be noted, however, that de Freitas Silva and co-workers detected signs of overconfidence in a questionnaire-based study conducted among 331 Magic the Gathering players in Brazil, “with 22.7% of players expressing confidence in their ability to identify cards with appreciation potential and 16.9% believing that their assessments are more accurate than those of specialized sellers” [90]. The authors concluded that “this overconfidence bias may lead players to underestimate the risks involved in card purchases, resulting in imprudent decisions, particularly in speculative markets” [90].

Holofoil cards fetched approximately seven times higher sales prices than matte cards (median 13.03 € vs. 1.85 €) and showed 4.5 times lower survival times (median 17 days vs. 78 days), corresponding to a 218% increased chance of being sold during the study period compared to matte cards according to the multivariable Cox regression analysis. Taken together, Rare and holofoil cards contributed overproportionately to the total revenue due to high median sales prices and rapid sales kinetics. In addition, the ABC analysis [77–79] showed that “A” cards (i.e., the most valuable 42% of all cards sold (93/220), which accounted for 80% of the total revenue) contained 43.0% (40/93) Rare and 24.7% (23/93) holofoil cards, in contrast to intermediate-value “B” cards, which only comprised 2.2% (2/84) Rare and 1.1% (1/84) holofoil cards, and the least valuable “C” cards, which did not contain Rare nor holofoil cards. Uncommon cards exhibited an interesting economic potential because they sold remarkably fast (albeit at relatively low prices). Besides the Pokémon TCG, pronounced price gaps regarding different grades of rarity, as well as between holofoil and matte cards, have also been observed for Magic the Gathering [26,84]. Specifically, Hughes showed that prices for Magic the Gathering cards in the highest rarity category (i.e., Mythic Rare) were 70–90 times higher than cards in the Common category, and that prices for foil versions were 2–4 times higher compared to non-foil cards [84].

In a field experiment conducted in April 2000, Katkar and Reiley investigated the impact of public versus secret reserve prices on auction outcomes on eBay using Pokémon trading cards [10]. While their results are not directly comparable to our findings since no auctions were conducted in our study, several aspects are noteworthy. Of 50 matched pairs of Pokémon trading cards “with values high enough to attract bidder interest on eBay” [10] (i.e., 100 cards in total), Katkar and Reiley were able to sell 59% (59/100) compared to 73.3% (220/300) in our study. Besides our sample being three times larger, the composition of the samples differed markedly, for example, the proportion of holofoils was 24% (24/100) in the Katkar and Reiley study [10] vs. 9.3% (28/300) in our investigation. In addition, the proportion of promotional cards was 8% (8/100) [10] vs. only 0.3% (1/300). The mean revenue per card (± SD) achieved by Katkar and Reiley was US$2.74 ± 1.65 [10] (corresponding to 4.40 ± 2.65 € in 2025, taking inflation [87] and the current exchange rate [88] into account) vs. 4.20 ± 10.38 € in our study. While the means were remarkably similar in both studies, the greater SD in our analysis can be explained by the more heterogeneous sample and potentially also by the high degree of quality uncertainty for buyers given the missing professional evaluation of our cards by an independent grading service [91].

Since their inception in 1995, online auctions have become an interesting and evolving field of economic research [86,92], a comprehensive discussion of which would go beyond the scope of this manuscript. In brief, different auction models (e.g., the independent private values (IPV) model, the common values (CV) model or the affiliated values (AV) model) and auction mechanisms exist (e.g., English or ascending-price auctions, Dutch or descending-price auctions, first-price sealed-bid auctions, second-price sealed bid or Vickrey auctions) [28,32,33,39,93–95]. Previous research indicated that certain selling strategies (e.g., the use of a starting bid or reserve price) may lead to higher auction outcomes and may influence price fairness perceptions [28,33,34,83,86,91]. The influence of bid shading, the phenomenon of the “winner’s curse” (where the winner of an auction overpays for the item in question) as well as the influence of seller reputation are other intriguing aspects of (online) auctions [28,32,91,94,95]. Higher seller reputation has generally been associated with greater seller revenue [28,31,35–37,86,95], even though not all existent data support this proposition. In studies by Resnick and Zeckhauser [96] and by Jin and Kato [85], for example, seller reputation did not influence auction prices; yet, higher seller reputation did increase the probability of a sale [85,91,96]. Moreover, platform effects, which describe the relationship between the auctioneer and the seller (e.g., price guarantees, commissions, buy-in penalties), should be taken into consideration (reviewed in [34]; for a contemporary and critical view on platform economics see also [97]). It will be exciting to explore in the future which auction strategy (from the seller’s perspective) yields the highest revenue if applied to the Pokémon TCG secondary market and how seller reputation and platform effects impact on auction prices. As the present study focused on posted-price transactions rather than auction dynamics, we will reserve the investigation of these topics for other researchers. An additional aspect that warrants consideration in this regard is that, under auction settings, the market independently determines the price. In the present analysis, by contrast, the posted prices acted as a ceiling on the possible sales prices by truncating the demand curve from above so that the cards’ “true” market-clearing prices could not be investigated with the applied methodology. This represents a substantial limitation of our study, which should be taken into consideration when devising future investigations.

Condition and edition of Pokémon trading cards did not significantly influence sales prices in our investigation. The former finding stands in contrast to a rule of thumb for Magic the Gathering cards put forward by Cavalletti and embraced by Weber, stating that when a card’s condition is decreased by one category (e.g., from Excellent to Very Good) its price usually decreases by at least 5–10% [26,98]. Physical condition has also been described as a critical factor in determining the value of sportscards, delimiting collectibles from traditional forms of investment like stocks or bonds [99]. In our study, cards from the unlimited edition displayed a 304% increased chance of being sold during the study period compared to first-edition cards according to the multivariable Cox regression analysis. However, this result should be interpreted with considerable caution due to the small number of first-edition cards contained in our sample (only 6 of 300 cards (2%)). A modeling artifact as an alternative explanation appears a reasonable possibility. In survival models, sparse categories with few events can lead to unstable coefficient estimates, with hazard ratios and confidence intervals that are highly sensitive to small data perturbations or random sampling variation. A single sale or non-sale can substantially shift the estimated hazard ratio and its confidence interval, increasing susceptibility to model instability and potential overfitting. This phenomenon is further exacerbated when several correlated covariates are included simultaneously, as in the present multivariable setting, which can inflate standard errors and increase the risk of spurious or exaggerated effect sizes for sparse categories. Future studies could mitigate this limitation by enlarging the sample size of first-edition cards or by applying penalized regression approaches (such as Firth’s penalized likelihood method) or related shrinkage methods that are specifically designed to stabilize estimates in the presence of sparse data. While the proportional hazards assumption of our Cox regression model was checked visually by assessment of Kaplan–Meier curves and log-minus-log plots, more advanced diagnostic tests such as the Schoenfeld residual test or sensitivity analyses to test the robustness of the model were not performed. In light of these methodological limitations, the results from our Cox regression model should be considered as exploratory, hypothesis-generating and preliminary. Importantly, sellers should not derive any direct practical or behavioral implications, specifically with regard to the large edition-related HR, from the model.

While Hiersemann described that Pokémon trading cards in mint condition and first-edition cards fetched the highest prices at auctions, his analysis focused on the TCG’s most valuable items (so-called “holy grails” like the Pikachu Illustrator card) [19]. The findings of our study, by contrast, potentially suggest that those considerations cannot be transferred one-to-one to the “ordinary” online secondary market for Pokémon trading cards. Based on the results of our investigation, it can be inferred that lay sellers should not be discouraged if they strive to sell cards in below-mint condition or cards from the unlimited edition on eBay (while keeping in mind the methodological constraints of the Cox regression model stated above). Whereas cards in better condition did not fetch higher median sales prices, we did however observe that cards in Excellent and cards in Near Mint or Better condition displayed 212% and 260% increased chances of being sold compared to cards in Very Good condition according to the multivariable Cox regression analysis.

A holofoil Charizard card in German language (Glurak 4/102 from the Base Set) was the most expensive card in our sample and alone contributed 15.7% (145.00 €/923.60 €) to the total revenue. Holofoil Charizard cards generally belong to the most famous and most valuable Pokémon trading cards [80], especially those from the first edition from 1999 [19]. Charizard has always been a favorite Pokémon character among aficionados, and the limited availability of the first edition further increases demand [19]. Cards in perfect condition have fetched prices of up to US$369,000 [14,18]. Our Charizard card was not from the first but from the unlimited edition and displayed considerable ultraviolet (UV) light damage in the upper quarter (color fading) [100]; otherwise the card could have been worth up to 600 € instead of 145 € (personal expert communication [101]). This example illustrates the importance of UV light protection to maintain the esthetic but also the economic value of trading cards [100].

Besides Charizard, there are also other iconic Generation I Pokémon, such as Pikachu and Squirtle. Pikachu and Squirtle have been characterized as Adler-type [102] superstar Pokémon by Gergaud and Verardi, i.e., Pokémon with comparatively high card prices despite inferior talent (defined as the aggregate measure of strength (expressed in damage points) and resistance (expressed in health points)) [80]. Gergaud and Verardi have explained this with Pikachu’s and Squirtle’s immense popularity among Pokémon fans since their appearance in “Pokémon: The First Movie”, a commercially very successful animated film which debuted in the United States in November 1999 [80,103–105]. According to the authors, collectors are willing to pay relatively large sums for Pikachu and Squirtle cards to establish and share a common fan culture rather than because of the cards’ gameplay utility [80]. Even though sales prices on Pokémon character level were not at the center of our investigation, an exploratory descriptive analysis supported the idea of Pikachu and Squirtle being Adler-type superstars [80,102]: While the median sales price of all cards in our study was 1.95 €, the median sales prices of nine Pikachu cards (four times the Base Set card 58/102, one Base Set 2 card 87/130, two times the Jungle card 60/64, and two times the Neo Genesis card 70/111) and four Squirtle cards (two times the Base Set card 63/102, two times the Team Rocket card 68/82) lay markedly higher, namely at 2.95 € and 3.85 €, respectively.

While sales prices did not differ significantly across the Pokémon trading card sets contained in our sample, univariable and multivariable Cox regression analyses consistently showed higher chances of being sold for Team Rocket and Gym Challenge cards compared to Base Set 2 cards (+98% and +249%, respectively, according to the multivariable model). The set Team Rocket had introduced Dark Pokémon to the TCG [7,51] and the Gym Challenge expansion had continued the popular concept of Owner’s Pokémon initially introduced by Gym Heroes [52–54] with new Pokémon Trainers (e.g., Giovanni and Koga) [55,56]. The Base Set 2, by contrast, essentially consists of reprints from earlier sets (Base Set and Jungle), which may explain why Base Set 2 cards were less popular in the present study. It can be speculated that collectors prefer originality to mere recapitulation, in line with a preference for variety, an important facet of behavioral economic theory [86–88]. From a psychological and social psychological viewpoint, Spaid reviewed possible motivations for collecting behavior, such as self-relevance (collecting as a personal challenge), social relevance (achieving expert status in a specific field of collectibles, prestige, and maintaining social relations with other collectors), hedonic benefit (leisure and esthetics), competition, need for uniqueness, brand attachment, historical preservation and continuity (preserving objects from the past to the future) and achieving immortality through one’s collection [22]. Importantly, the author differentiates between “serious” collectors who engage in the act of collecting with passionate endeavor from “acquisitive” collectors who merely collect for investment but are little interested in the type of object they collect [22]. Carey notes that set completion is another major driving force in the collecting process, and that financial and non-financial motives of collectors are not mutually exclusive since “a complete set may be worth more in the secondary market, if one exists, than the sum of the individual pieces. This reflects the value of the rarest pieces and the opportunity costs of obtaining them.” [23]. Furthermore, nostalgia has been described as an important motivating factor along the entire collecting journey, promoting the beginning of a collection, influencing the progress of a current collection, and being a barrier to ending a collection [106,107]. Nostalgia plays a decisive role in the Pokémon TCG market, with many collectors having begun their collecting journey in childhood or adolescence and continuing it into adulthood [3,13]. Lee et al. describe collecting as a form of (experiential) consumption and comprehensively review the theoretical framework applied in the existent literature to explain collecting behavior [107]. From a managerial point of view, companies should consider that nostalgia can influence product consumption in a circular manner [106], as can be observed in the Pokémon TCG market, which experienced its climax in the late 1990s, a declining interest from the mid-2000s onwards [3,12], and a remarkable comeback during the 2020s [4,13]. The latest chapter in this development was the spectacular sale of a PSA 10-graded Pikachu Illustrator card owned by wrestling star and social media personality Logan Paul to venture capitalist AJ Scaramucci at an auction conducted on 16 February 2026 for US$16.492 million (together with the diamond necklace Paul wore to present the card to the public for the first time in 2022), the highest price ever achieved for a trading card [15].

According to a survey of 175 players across 14 English-based online TCG message boards in May 2009, 84.1% of players who identified their gender were male (numerator and denominator not reported) [9]. Similarly, in a questionnaire study among Magic the Gathering players in Brazil, 92.1% (305/331) of participants were male [90]. In our study, Pokémon trading card buyers were also predominantly male (91.8% (202/220)), but on average female buyers spent 30% more money per trading card than male buyers (5.35 € vs. 4.12 €). It might be hypothesized that female collectors purchase fewer items overall but tend to invest more in each card, focusing on quality and rarity rather than quantity, resulting in higher per-item expenditures. Male buyers, on the contrary, might strive for higher volume or broader collections, which can dilute per-item spending. A similar trend (albeit involving much larger sums) has been noted in purchasing fine art, decorative art, and antiques, where median expenditures of female collectors constantly exceeded those of their male counterparts according to the Art Basel and UBS Survey of Global Collecting in 2023 [108]. For a detailed analysis of the portrayal of gender roles in the Pokémon animated TV series see [109].

The European TCG retail market is similar in size as the United States (market volume of around US$800 million in 2008), and Germany has historically been among the top five European Union markets, together with France, Italy, Spain and the United Kingdom (before Brexit) [9]. In the present study, the German federal states Thuringia and Hamburg emerged as Pokémon trading card hubs, displaying consistently positive economic prospects with regard to numbers of cards sold and cumulated revenues. Both federal states, especially in urban areas and university cities like Erfurt and Jena (Thuringia), have a strong concentration of younger populations, including students and young professionals who are key demographics for Pokémon card collecting and gameplay. While in our study no information on buyers’ age was available, the average Pokémon TCG player is 22 years old according to a survey by David-Marshall and colleagues [9]. Regular league meetings and vibrant local communities may encourage collecting and trading, which is especially visible in Erfurt, where an active Pokémon League organizes play and exchange events [110]. Hamburg, as a large metropolitan area, hosts more specialty shops, events, and conventions, with the regular trading card convention being a catalyst for a lively trading and collector scene [13,111,112]. In addition, Hamburg enjoys one of Germany’s highest per-capita income levels, meaning collectors have more disposable income to spend on hobbies such as the Pokémon TCG [113,114].

On the other hand, Hesse, Saxony and Berlin exhibited less favorable economic environments for Pokémon trading card sales in the present investigation. Economic prospects were potentially favorable for Bavaria and Brandenburg, but also North Rhine-Westphalia, Baden-Wuerttemberg and Lower Saxony should not be neglected due to their large population sizes [73], with numbers of cards sold and cumulated revenues being approximately proportionate to the number of inhabitants.

The proportions of cards sold as well as the proportions of the total revenue tended to increase with the population size of buyers’ residence, being higher in urban compared to more rural settings. By contrast, latencies between offer and sale of Pokémon trading cards tended to be shorter in rural than in urban locations. This might be explained by the higher density of trading card offers in urban surroundings. Besides eBay and other e-commerce platforms, potential buyers might also search locally, for example, via the app *Kleinanzeigen*, which is very popular in Germany [115,116]. Hence, potential buyers in urban centers might find more possibilities to compare price offers and look for the cheapest price, also by circumventing shipping costs if purchased items are fetched in person from vendors. To conduct such research for the cheapest price, potential buyers would however have to spend additional time, leading to higher latencies.

Whereas cards in German language and non-German cards sold at similar prices, the median survival time of German cards was almost three times shorter compared to non-German cards (48 days vs. 134 days), potentially due to a lower language barrier for buyers residing in Germany. Easier recognizability and understandability of cards in mother tongue (or primary language spoken) might be important aspects for buyers who acquire Pokémon trading cards not only as an investment but also for their original purpose, that is, playing the TCG.

In the beginning of the Pokémon TCG in the late 1990s and early 2000s, vendors and interested collectors had to retrieve market values of trading cards manually from paper-based compendia [117,118], gaming magazines [119] or needed to inquire current market prices in person at card stores [10,80]. This changed fundamentally with the increase in online search capabilities (e.g., through freely accessible search engines like Google [120] or Bing [121]), specialized online marketplaces (e.g., Cardmarket [122] or TCGplayer [123]), and—more recently—the advent of AI applications like ChatGPT [124], Gemini [125] or Perplexity [29]. In the present study, we found that Perplexity displayed potential utility in estimating realistic market price ranges for Pokémon trading cards in 60.7% (133/219) of cases when a hybrid workflow and a posted-price strategy were adopted, in which Perplexity was used as a decision support tool but the human seller remained in charge of the decision-making process. The applied methodology was selected in order to reflect real-world conditions for non-professional sellers on the Pokémon TCG secondary market, where we expected human sellers not to put unreserved trust in AI tools. The proportion of sales prices that lay below the price ranges initially indicated by Perplexity was higher than the proportion of cases in which sales prices above the Perplexity range could be fetched, suggesting strong competition in the Pokémon trading card business. When only cards for which the AI price range suggestions had been adopted for posted prices in the first place were considered, Perplexity price ranges could be achieved in 87.2% (123/141) of cases. Importantly, in the context of the present analysis, exogenously fixed posted prices acted as a ceiling on possible sales prices by truncating the upper segment of the demand curve, in contrast to auctions, where prices are endogenously determined by market forces. Therefore, the cards’ underlying (“true”) market-clearing prices could not be identified with the applied methodology, which constitutes a substantial limitation of our study.

To date, information about the utility of AI tools in the collectibles market is sparse but promising results have been obtained for antiques, where the integration of AI models—specifically convolutional neural networks (CNNs)—in the authentication process demonstrated impressive accuracies (94–96%) on the e-commerce platform Antique Hub [126]. Encouraging results were also obtained in the field of numismatics, where CNNs showed accuracies of 86.9% for country classification and 96.4% for value classification [127]. For TCGs, Curti Sanches showed that advanced machine learning techniques have the potential to outperform traditional statistical methods in forecasting prices of Magic the Gathering cards [128]. Kader and Lee recently investigated the performance of reasoning large language models (LLMs; e.g., OpenAI-o1) in three classical games from behavioral economics [129]. The authors showed that reasoning LLMs exhibited superior strategic reasoning capabilities compared to standard LLMs (e.g., ChatGPT-4), and often matched or exceeded human performance [129]. Their findings bear important implications for the future application of AI systems in economics, for example, in investment and trading [129]. Regarding alternative asset classes, machine learning techniques have also been applied to predict the price development of cryptocurrencies like Bitcoin, but several challenges and important questions about forecasting accuracy remain [130].

Whether collectibles qualify as legitimate investments has been long and hotly debated as they lack intrinsic worth and do not generate income like real estate, stocks, or bonds [26,81]. Their value depends solely on what potential buyers are willing to pay, making them highly volatile and vulnerable to changing trends [26,81]. From a financial investment perspective, collectibles often perform less favorably compared to traditional asset classes [22,107,131]. The collectibles market can be characterized as a thin market [28,131]. Thin markets typically comprise “items that are more heterogeneous across key attributes and are of varying quality levels; some examples are used furniture and rare antiques. In a thin market it is more difficult to identify a fair market price because less information is available, and because seemingly similar items can vary considerably in terms of their underlying attributes and quality.” [91]. Hou and Blodgett have highlighted that the importance of seller and buyer expertise is strongest in thin markets with a high level of quality uncertainty [91], which applies to the Pokémon TCG secondary market. Due to the greater heterogeneity of items in thin markets, buyers tend to act more cautiously. In thin markets, seller expertise can serve as an “indicator of non-opportunism and trustworthiness” [91], and it is expected that higher degrees of seller expertise reduce risk and exert a more pronounced influence on the price than in thick markets (i.e., markets “in which relatively homogeneous items are auctioned on a regular basis” [91] and in which “it is relatively easy for buyers to identify a fair market price because of the availability of information about what others have recently paid for similar or identical items” [91]). With respect to investing in the collectibles market, it is generally agreed that the type of collectible matters, for example, fine art is valued more seriously than fad-driven items like trading cards [26,81,107]. However, advancements in technology have reshaped the collectibles market, especially for trading cards [26,81]. Online platforms like eBay have lowered costs, reduced barriers and information asymmetries, and made buying and selling more efficient, resulting in a more unified and transparent marketplace [26,81]. Technology has enabled easier access to historical data, improving transparency, analysis, and valuation [26,81]. These changes may help trading cards gain recognition as an interesting alternative investment class to diversify portfolios [16–18,26,81,82]. Over the past 20 years, the performance of Pokémon trading cards has exceeded that of the Standard & Poor’s (S&P) 500 (a stock index that combines the 500 strongest stock exchange-listed US companies) [16,17]. While the value of Pokémon trading cards increased by 3261% over the last two decades, the S&P 500 rose by 421% [17]. Similar methodological approaches have been used to estimate rates of return of different sports card portfolio strategies in the past [98], but also more recently [132,133]. Hilbert, for example, compared the investment into sports cards (baseball, basketball, football) and non-sports cards (Pokémon) with the S&P 500 and a bond index (Treasury Bills) over a three-year period (January 2021 till December 2023), and found that collectibles portfolios underperformed traditional asset classes. Specifically, Pokémon trading cards returned –4.72% annually over the selected time period, leading the author to conclude that “card prices in the short-term are basically random” [132,133]. In the future, taking advantage of AI applications like deep neural networks may optimize portfolio management of alternative asset classes like Pokémon trading cards [82]. The speculative nature of such a form of investment should however not be neglected, especially in view of younger generations’ increasing preference for high-risk asset classes [134]. While some collectors may achieve extraordinary profits, others have been incurring serious amounts of debts [17].

The financial behaviors surrounding the acquisition of trading cards may overlap with patterns of psychological and behavioral risk [20,135,136]. Practices such as compulsive purchasing, hoarding, and dependence on the thrill of pulling rare cards from booster packs share characteristics with gambling and addictive behaviors [107,137], even though not all available data point into this direction [11]. It therefore appears prudent to consider potential mental health-related implications of the Pokémon TCG. Exploring such dimensions is important because consumer decisions in the collectibles markets are not solely driven by rational economic incentives but may also be shaped by underlying cognitive biases, reward-processing mechanisms, and mental health vulnerabilities [107]. Hence, with our professional background as physicians, we searched the medical literature for potential cross-links with the Pokémon TCG to provide a more holistic perspective, linking market dynamics with public health implications. To this end, the literature database PubMed/MEDLINE [138] was searched on 13 August 2025 for relevant articles, using the search string “(pokémon OR pokemon) AND (psychiatry OR gambling OR illness OR dependence OR dependency OR compulsive hoarding OR hoarding)”. The results of our literature search are reported in narrative form in the following.

Besides Pokemon (spelled without accent) being a transcription factor and proto-oncogene whose overexpression is linked to the development of several human cancer types (especially B- and T-cell lymphomas) [139,140], medical research has mainly focused on the effects of the mobile augmented reality game Pokémon GO [141] on both physical [142,143] and mental health [144,145]. While the effects of Pokémon GO on human players have overall been described as beneficial (e.g., increased physical activity and less sedentary behavior [142,143,146], reduced social withdrawal [147,148], higher self-reported social functioning and life satisfaction [145]), other authors shed light on Pokémon GO-related traumatic injuries due to falls or traffic accidents when players were distracted and inattentive to their surroundings [149]. Also, the placement of Pokémon characters in medical settings (e.g., emergency departments, hospital wards, etc.) has raised concerns about potential breaches of confidentiality [150].

An older report analyzed an outbreak of mass psychogenic illness among more than 12,000 Japanese children who displayed symptoms resembling photosensitive epilepsy (such as convulsions, loss of consciousness, and nausea) after episode 38 of the Pokémon animated TV series termed *Dennou Senshi Poligon* (“Computer Warrior Polygon”) had aired in December 1997 [1]. Yet, we did not retrieve any studies that specifically addressed medical implications of the Pokémon TCG. Notwithstanding, there have been reports of thefts, assaults, bullying, and obsessive behaviors in connection with Pokémon trading cards, even among children [12–14,66]. This had led to Pokémon trading cards being banned from some schools in the 1990s [12], but also more recently [13].

The present investigation has several limitations. First of all, the Pokémon trading cards in our sample were privately owned and even though nine different sets, four different languages and three different condition categories were represented, the proportions of the sets, languages and conditions were not evenly distributed, limiting the generalizability of our results. Moreover, the cards were not graded professionally by an independent third-party service like PSA [24] or BGS [25], a limitation our study shares with the study by Katkar and Reiley [10]. The condition categories were self-assessed by a single evaluator (JH), introducing potential measurement error and quality uncertainty for potential buyers [85,91]. Of note, Jin and Kato detected that buyers were willing to pay 27% more for cards that were self-assessed as 9 to 9.5 (i.e., mint condition) and 47% more for cards that were self-reported as 10 (i.e., gem mint condition) [28,85]. Since there was only one evaluator in our study, an interrater reliability was not determinable. It is known that professional grading significantly influences secondary market value [5,85]. Possibly, different results might have been obtained if the cards in our sample had been graded by an independent third-party service. However, professional grading costs about US$25 per card [26], which was not considered economically feasible for 300 cards in the present study, which was investigator-initiated and which did not receive any external funding.

In our study, the AI tool Perplexity was applied from the user’s perspective as a decision support tool to reflect real-world conditions for non-professional sellers. The underlying AI model itself, however, was not part of the investigation; hence, no performance metrics such as error measures or calibration analyses were conducted. Such an analysis would have been beyond the scope of the present study but should definitely be performed in the future, preferably by including the additional expertise of computer scientists. In addition, future studies should adopt a workflow in which Perplexity price ranges are not overridden by human sellers in order to more accurately assess Perplexity’s independent utility in estimating sales prices of Pokémon trading cards. Also, study designs are conceivable in which human sellers with and without AI decision support, or even different AI tools (such as Perplexity versus ChatGPT) compete against each other to maximize revenues. Moreover, future studies should preferably be conducted under auction settings in order to more accurately identify “true” market-clearing prices of Pokémon trading cards.

The present work focused exclusively on the sale of physical trading cards from the traditional Pokémon TCG. Digital adaptations like Pokémon TCG Live [4,151] or the recently launched Pokémon TCG Pocket [152] were not part of the analysis. Moreover, the focus of our study was primarily economic (with a brief excursion to the medical literature), but not psychological. We did not evaluate factors that might have motivated buyers to purchase trading cards, nor did we assess other biopsychosocial determinants (beyond gender and residence) that might have affected purchasing behavior.

An additional limitation of the present investigation is that gender was not self-identified but was derived from buyers’ first names serving as surrogate parameter [70]. In one case, only the initial of the buyer’s first name was provided; this was considered as gender not determinable. To maintain data integrity, we excluded this case from inferential statistics that explored potential gender-specific differences. Future studies should preferably adopt a gender classification scheme that is based on buyers’ self-identification.

Future research on Pokémon trading cards should also take more recent expansions of the TCG into consideration, such as the e-Card [153], EX [154] and Diamond & Pearl series [155]. In the present study, the conventional three-tier rarity classification (Common; Uncommon; Rare) was used. Future investigations should consider analyzing Pokémon trading cards of advanced rarity subcategories such as Shiny Rare, Double Rare, or Ultra Rare [156]. In addition to standard holofoil cards, also reverse holofoil cards should be studied in the future. In reverse holofoil cards, the holographic effect is applied to the card’s background, whereas the main Pokémon image remains non-shiny [157].

## Conclusions

The economic value of Pokémon trading cards, particularly as collector’s items, underscores their significance beyond mere playthings, making them a subject of both academic and commercial interest. In this prospective one-year field study, sales prices of Pokémon trading cards showed a markedly skewed distribution. Rare and holofoil cards contributed overproportionately to the total revenue. Uncommon cards exhibited an interesting economic potential due to rapid sales kinetics. Team Rocket and Gym Challenge cards were more popular than Base Set 2 cards, as reflected by shorter survival times. The vast majority of buyers were male, but on average female buyers spent more money per trading card. The German federal states Thuringia and Hamburg emerged as Pokémon trading card hubs. Pokémon trading cards appear as an interesting alternative asset class to diversify portfolios but due to the speculative nature of the collectibles market, caution should be exercised. Important limitations of our study include a narrow sample of privately owned Pokémon trading cards from select and unevenly distributed sets, languages and conditions, a low proportion of high-value items, potential misclassification of card condition due to missing professional grading, as well as several methodological (e.g., use of posted prices instead of auction mechanisms) and statistical constraints (e.g., potential instability introduced into the multivariable Cox regression model by sparse categories), which may have led to systematic biases that limit the extent to which our results can be generalized to the broader Pokémon market or to other TCGs. Even though our review of the medical literature did not reveal negative effects of the Pokémon TCG on mental health, future research should explore this topic more extensively, especially since Pokémon trading cards are immensely popular among children, adolescents and young adults, who might be particularly psychologically vulnerable.

## Supporting information

S1 Dataset Convenience sample of 300 privately owned Pokémon trading cards offered on the e-commerce platform eBay between 28 May 2024 and 27 May 2025, 220 of which were sold during the study period. The cards are listed in order of descending sales prices.
(XLSX)

S2 Table Selection of five out of 300 Pokémon trading cards offered on the e-commerce platform eBay, 220 of which were sold during the study period. The depicted cards represent the minimum, first quartile, median, third quartile, and maximum of all sales prices.
(DOCX)

S3 Table Pokémon trading card sales and revenue potentials of the 16 German federal states. (DOCX)

## Abbreviations

AI: artificial intelligence
AV: affiliated values
BGS: Beckett Grading Services
*p*_adj_: adjusted *p*-value
CI: confidence interval
CNN: convolutional neural network
COVID-19: coronavirus disease 2019
CV: common values
€: euro
e-commerce: electronic commerce
GPT: generative pre-trained transformer
HR: hazard ratio
IPV: independent private values
IQR: interquartile range
n.a.: not applicable
n.e.: not estimable
no.: number
PP: percentage point
PSA: Professional Sports Authenticator
SD: standard deviation
S&P: Standard & Poor’s
TCG: trading card game
TV: television
USA: United States of America
US$: United States dollar
UV: ultraviolet
WWE: World Wrestling Entertainment.
