# Supplementary material for: Sales characteristics of Pokémon trading cards: A prospective one-year field study
Source: PLoS One. 2026 Mar 12;21(3):e0334289. doi: 10.1371/journal.pone.0334289 (PMC12981466; doi:10.1371/journal.pone.0334289)
Supplement: S2 Table — The depicted cards represent the minimum, first quartile, median, third quartile, and maximum of all sales prices. (DOCX) [file pone.0334289.s002.docx]

**S3 Table.** **Pokémon trading card sales and revenue potentials of the 16 German federal states.**

| **Federal state** | **No. of inhabitants (as of 31 December 2023)** | **% of German population** | **No. of cards sold** | **% of all cards sold** | **Pokémon trading card sales potential; Δ (PP)** | **Cumulated revenue (€)** | **% of total revenue** | **Pokémon trading card revenue potential; Δ (PP)** |
| --- | --- | --- | --- | --- | --- | --- | --- | --- |
| North Rhine-Westphalia | 18,190,422 | 21.5 | 52 | 23.6 | ↔ ; 2.2 | 179.70 | 19.5 | ↔ ; –2.0 |
| Bavaria | 13,435,062 | 15.9 | 50 | 22.7 | ↑ ; 6.9 | 150.17 | 16.3 | ↔ ; 0.4 |
| Baden-Wuerttemberg | 11,339,260 | 13.4 | 24 | 10.9 | ↔ ; –2.5 | 78.70 | 8.5 | ↘ ; –4.9 |
| Lower Saxony | 8,161,981 | 9.6 | 21 | 9.5 | ↔ ; –0.1 | 91.69 | 9.9 | ↔ ; 0.3 |
| Thuringia | 2,122,335 | 2.5 | 19 | 8.6 | ↑ ; 6.1 | 69.70 | 7.5 | ↗ ; 5.0 |
| Schleswig-Holstein | 2,965,691 | 3.5 | 15 | 6.8 | ↗ ; 3.3 | 21.60 | 2.3 | ↔ ; –1.2 |
| Hamburg | 1,910,160 | 2.3 | 14 | 6.4 | ↗ ; 4.1 | 50.50 | 5.5 | ↗ ; 3.2 |
| Rhineland-Palatinate | 4,174,311 | 4.9 | 5 | 2.3 | ↘ ; –2.7 | 49.05 | 5.3 | ↔ ; 0.4 |
| Hesse | 6,420,729 | 7.6 | 4 | 1.8 | ↓ ; –5.8 | 13.65 | 1.5 | ↓ ; –6.1 |
| Saarland | 994,424 | 1.2 | 4 | 1.8 | ↔ ; 0.6 | 8.49 | 0.9 | ↔ ; –0.3 |
| Berlin | 3,782,202 | 4.5 | 3 | 1.4 | ↘ ; –3.1 | 16.00 | 1.7 | ↘ ; –2.7 |
| Brandenburg | 2,581,667 | 3.0 | 3 | 1.4 | ↔ ; –1.7 | 161.90 | 17.5 | ↑ ; 14.5 |
| Saxony-Anhalt | 2,180,448 | 2.6 | 2 | 0.9 | ↔ ; –1.7 | 4.35 | 0.5 | ↔ ; –2.1 |
| Mecklenburg-Vorpommern | 1,629,464 | 1.9 | 2 | 0.9 | ↔ ; –1.0 | 24.10 | 2.6 | ↔ ; 0.7 |
| Saxony | 4,089,467 | 4.8 | 1 | 0.5 | ↘ ; –4.4 | 3.00 | 0.3 | ↘ ; –4.5 |
| Bremen | 691,703 | 0.8 | 1 | 0.5 | ↔ ; –0.4 | 1.00 | 0.1 | ↔ ; –0.7 |

Pokémon trading card sales and revenue potentials of the German federal states were calculated as follows:

- **↑** : difference Δ between the proportion of all cards sold or the proportion of the total revenue and the proportion of the German population living in the respective federal state > 5.0 percentage points (PP)
- **↗** : 2.5 PP < Δ ≤ 5.0 PP
- **↔** : –2.5 PP ≤ Δ ≤ 2.5 PP
- **↘** : –5.0 ≤ Δ < –2.5 PP
- **↓** : Δ < –5.0 PP

Abbreviations: no., number; PP, percentage point
